# Validation of an Automated Wearable Camera-Based Image-Assisted Recall Method and the 24-h Recall Method for Assessing Women’s Time Allocation in a Nutritionally Vulnerable Population: The Case of Rural Uganda

**DOI:** 10.3390/nu14091833

**Published:** 2022-04-27

**Authors:** Andrea L. S. Bulungu, Luigi Palla, Jan Priebe, Lora Forsythe, Pamela Katic, Gwen Varley, Bernice D. Galinda, Nakimuli Sarah, Joweria Nambooze, Kate Wellard, Elaine L. Ferguson

**Affiliations:** 1 Department of Population Health, London School of Hygiene and Tropical Medicine, London WC1E 7HT, UK; bernicegalinda9@gmail.com (B.D.G.); kigozi1995@gmail.com (N.S.); elaine.ferguson@lshtm.ac.uk (E.L.F.); 2 Department of Public Health and Infectious Diseases, University of Roma La Sapienza, 00185 Rome, Italy; 3 Department of Medical Statistics, London School of Hygiene and Tropical Medicine, London WC1E 7HT, UK; 4 School of Tropical Medicine and Global Health, University of Nagasaki, Nagasaki 852-8102, Japan; 5 Natural Resources Institute (NRI), University of Greenwich, Chatham Maritime, Kent ME4 4TB, UK; jan.priebe@gmail.com (J.P.); l.forsythe@greenwich.ac.uk (L.F.); p.g.katic@greenwich.ac.uk (P.K.); gwendolyn.varley@slu.se (G.V.); k.wellard@greenwich.ac.uk (K.W.); 6 Africa Innovations Institute (AfrII), Kampala P.O. Box 34981, Uganda; jngalabuzi@yahoo.com; 7 Department of Nutritional Sciences and Dietetics, Kyambogo University, Kyambogo, Kampala P.O. Box 1, Uganda

**Keywords:** validation studies, time use, methodology, wearable camera, measurement error, care practices, maternal time

## Abstract

Accurate data are essential for investigating relationships between maternal time-use patterns and nutritional outcomes. The 24 h recall (24HR) has traditionally been used to collect time-use data, however, automated wearable cameras (AWCs) with an image-assisted recall (IAR) may reduce recall bias. This study aimed to evaluate their concurrent criterion validity for assessing women’s time use in rural Eastern Ugandan. Women’s (*n* = 211) time allocations estimated via the AWC-IAR and 24HR methods were compared with direct observation (criterion method) using the Bland–Altman limits of agreement (LOA) method of analysis and Cronbach’s coefficient alpha (time allocation) or Cohen’s κ (concurrent activities). Systematic bias varied from 1 min (domestic chores) to 226 min (caregiving) for 24HR and 1 min (own production) to 109 min (socializing) for AWC-IAR. The LOAs were within 2 h for employment, own production, and self-care for 24HR and AWC-IAR but exceeded 11 h (24HR) and 9 h (AWC-IAR) for caregiving and socializing. The LOAs were within four concurrent activities for 24HR (−1.1 to 3.7) and AWC-IAR (−3.2 to 3.2). Cronbach’s alpha for time allocation ranged from 0.1728 (socializing) to 0.8056 (own production) for 24HR and 0.2270 (socializing) to 0.7938 (own production) for AWC-IAR. For assessing women’s time allocations at the population level, the 24HR and AWC-IAR methods are accurate and reliable for employment, own production, and domestic chores but poor for caregiving and socializing. The results of this study suggest the need to revisit previously published research investigating the associations between women’s time allocations and nutrition outcomes.

## 1. Introduction

Time is an essential resource for caregiving, including care for women, child feeding and psychosocial and cognitive stimulation, hygiene practices, home health practices, and food preparation and storage [1]. In poor households of low-income countries, the provision of essential needs (food, water, care) relies primarily on the time and labor of household members themselves. Longstanding evidence from time-use studies around the world indicates that, despite high rates of participation in productive work, the burden of care for children and other household members remains primarily with women [2,3,4,5,6]. Understanding the simultaneous demands on women’s time for basic survival, and the trade-offs made between time allocated to food production, food preparation, income-generating activities, home maintenance, and care of children and other members of the household, is essential for understanding the factors influencing nutrition in low-income country contexts [7,8,9,10,11,12,13].

Popkin (1980) first demonstrated that a mother’s time spent on childcare was positively associated with child nutrition status [14] and, subsequently, the importance of time for care has been well understood to be a key factor for maternal and child nutrition and overall well-being [1]. Yet, decades of empirical studies have shown the relationship between women’s time allocation and maternal and/or child nutrition to be complex. Gryoboski (1996) found that time allocated to childcare by aunts, sisters, and grandmothers was associated with increased caloric intake for children [15], whereas the association between the mother’s time allocated to childcare and caloric intake was negative, and Komatsu et al. (2018) found that the association between women’s time allocated to “reproductive work” and child nutrition depended on the economic status of the household [8]. Other studies have taken the opposite approach, exploring instead the influence of women’s time allocated to productive work on their and/or their child’s nutritional status. The results of these studies have also been conflicting. Some have found that a mother’s time allocated to (agriculture) work was negatively associated with child nutritional status [16,17], whereas others have found there to be no relationship between a mother’s time allocated to work and child nutritional status [12,18].

Previous studies suggest that the amount of time women allocate to both productive and reproductive work is severely undercounted [19,20,21,22,23,24,25], which limits our ability to accurately assess how women spend their time and its influence on women’s and children’s nutrition, health, and well-being [16,26,27,28,29,30,31,32,33]. The boundaries of “reproductive work” are not rigid. Caregiving responsibilities and other domestic chores tend to overlap with time allocated to income-generating activities or leisure. A large proportion of reproductive work, such as childcare, is performed while simultaneously performing other tasks [27,34,35]. Rather than time spent in “productive work” displacing time spent in “reproductive work”, women tend to manage their dual roles by simply working more hours [36] and/or multitasking [37,38,39]. This overlap in productive and reproductive work is differentially detrimental to measuring women’s time in their dual role as caregivers and income generators. Current methods for measuring time allocation have limitations that undermine efforts to accurately estimate simultaneous activities, especially in rural, low-income country contexts [27,34,40,41,42,43,44,45].

For over a century, surveys in high-income countries have typically utilized time-use diary methods (i.e., time budgets—either prospective or retrospective) and these are still considered to be reliable methods of assessing time use [46,47,48]. However, for low-income country contexts where literacy is low, or the sense of time does not align with western constructs, time-use diaries are infeasible [49]. In these contexts, direct observation is generally considered to be the “gold standard” for collecting time allocation data [6,22,50,51,52,53,54,55,56]. For time use, “direct observation” entails a researcher being at participants’ homes, watching and recording everything they do. It is resource intensive, requires specialized skill, is burdensome for the participant, and is prone to various forms of reactivity bias, including the Hawthorne effect, observer-expectancy effect, and observer bias [30,57,58,59]. Therefore, for time-use surveys in low-income countries, the 24 h recall (24HR) method is commonly used [40,51,55,60,61,62]. The 24HR method is a structured interview where the interviewer asks the respondent, for time use, about all the activities they performed the previous day.

Recall methods, such as the retrospective time-use diary, the 24 h “free” recall technique utilized in the time-use module of the Women’s Empowerment in Agriculture Index (WEAI), or the more conventional “stylized questionnaires”, levy a heavy cognitive burden on the participant and are prone to error and bias [63,64,65]. Their accuracy relies upon the respondent’s memory and motivation, as well as on the skill and persistence of the interviewer. Misreporting of activities may occur, either unintentionally or intentionally, due to biases (i.e., recall, interviewer, social desirability, or approval biases), low literacy or numeracy, or cognitive phenomena unique to the recall of time allocation, such as “telescoping” and the burden of aggregating time across hours of the day, days of the week, or seasons of the year [23,66,67,68]. In particular, recall methods poorly capture reproductive activities that are typically performed by women in rural low-income country contexts, such as: childcare and feeding, food preparation, domestic chores (fetching fuel, washing clothes, etc.), healthcare seeking, and socializing [50,69].

Historically, informal work (e.g., selling food stuffs in unregulated markets), such as is commonly performed by women in rural low-income countries, was unrecognized in large-scale labor force and time-use studies [19,21,27]. Efforts to remediate the under-reporting of informal and reproductive work have culminated in the launch, in 2019, of the International Classification of Activities for Time-Use Statistics (ICATUS-2016) [70]. These recent improvements, however, do not address the methodological limitations of traditional retrospective time allocation assessment methods for measuring work predominantly done by women in rural low-income country settings. 

Automated wearable cameras (AWCs) are inexpensive technologies that prospectively and unobtrusively record activities as they are performed. AWCs have been investigated as a research method in high-income countries for assessing diet [71,72,73,74,75,76,77,78,79,80,81,82,83], physical activity [84,85,86,87,88,89], and the food environment [90,91,92,93,94,95]. Only limited research with AWCs has been undertaken in low- or middle-income country contexts. They have been evaluated for assessing diet in Tonga [96], China [97], and Uganda [98], and childcare practices in South Africa and Nepal [99].

An IAR is a method using photographs, either automatically generated from an AWC or taken by the participant, as an autobiographical memory cue (recall trigger) to help respondents reconstruct key details from their previous day [100,101,102,103]. Only a few studies using an AWC-based IAR to assess time allocation have been conducted, all in the UK [102,104,105]. Bulungu et al. (2021) identified several challenges unique to rural low-income country settings which may affect their performance in assessing women’s time use, including a subject’s difficulty in interpreting the wearable camera’s first-person perspective photos, activities happening outside the camera’s field of vision, and poor lighting [98].

This study was, therefore, undertaken in rural Eastern Uganda to evaluate the concurrent criterion validity, for assessing women’s time use, comparing both an AWC-based image-assisted recall (IAR) method and the 24HR method to direct observation (criterion method). No study, to our knowledge, has examined the criterion validity, for assessing women’s time use in a low-income country context, of the 24HR method or an AWC-based method in either free-living or controlled settings. In addition, as described by Bulungu et al. (2021), in this study population the median dietary diversity score was 4 for both women (out of 10 food groups) and children (out of seven food groups), with only 41% and 42% of women and children achieving the minimum dietary diversity, respectively, which indicates this population represents a nutritionally vulnerable one.

## 2. Materials and Methods

### 2.1. Study Design

This study was nested within a cross-sectional study of women with a child aged between 12 and 23 months inclusive (*n* = 211), to examine the impact of a labor-saving technology on women’s time for childcare, food preparation, and dietary practices. The study was conducted between January and February 2018 in Bugiri and Kamuli Districts, Eastern Region, Uganda.

In our study, women’s time allocation was assessed, for the same day, using three concurrent methods: (1) direct continuous observation (15 h), (2) 24HR, and (3) IAR using photos captured via an AWC. An IAR is a method using photographs, either automatically generated from a wearable camera or taken by the participant, as an autobiographical memory cue (recall trigger) to help respondents reconstruct key details from their previous day [100,101,102,103]. 

Data were collected over five consecutive days, following one of two possible patterns, as presented in Supplementary Table S1. Specifically, for both patterns, on day 1, eligibility was confirmed, a structured questionnaire was administered, and anthropometric data were collected for all participants. For half of the study participants, on day 2, time allocation data were collected using direct observation and recorded on the AWC attached to the respondent. On day 3, a 24HR was administered, followed by an IAR using photos captured on day 2 by the AWC. On day 4, time allocation data were again recorded via AWC only (i.e., no observation). On day 5, an IAR was administered using photos captured on day 4 by the AWC. The other half of the study participants began with the AWC only (i.e., days 2 and 4 were switched) and ended with all three methods (i.e., days 3 and 5 were switched). For all participants, on the 5th day, a final structured questionnaire was also administered. Time allocation data collection was distributed across all days of the week at the population level to account for a day-of-the-week effect, and for each respondent, the enumerator assigned to conduct the direct observation was different from the enumerator assigned to administer the 24HR and IAR.

Ethical approval was obtained from the [location masked for blind review] (A24ES), [location masked for blind review] Research Ethics Committee (Project ID: 1420), and [location masked for blind review] Ethics Committee (Project ID: B0501). Following community sensitization, verbal explanation of the study, and demonstration of the AWC, written consent (signature or thumb print) was obtained from all respondents who participated in our study.

### 2.2. Participants and Sampling

Twenty-two villages were purposefully selected, for this study, of which eleven had access to labor-saving technology and eleven did not. These villages participated in the Sasakawa Global 2000 Uganda (SG2000 Uganda) country program (the local implementing partner for the parent study). The sample size calculation (*n* = 264; 22 communities, 12 households per community) was based on requirements of the main study within which this current study was nested. This sample size was deemed sufficient for the current validation study, using the Bland–Altman (BA) method of analyses [106,107,108].

The sampling frame, for each village, was a household listing of all women with a child born between 1 January 2017 and 1 May 2017 (children aged 12 to 23 months at the time of data collection). These lists were generated by the SG2000 community-based facilitators. Twelve mother–child dyads in each village were randomly selected to participate in the study. Substitutions were made, as needed, until 12 mother–child dyads who met the inclusion/exclusion criteria were recruited. Mother–child dyads were excluded if the child was less than 12 months or greater than 23 months of age, was not yet eating solid foods on a regular basis, or was a multiple-birth child; the mother was unable to communicate in Lusoga, Luganda, or English; either the mother or child had a severe disability; the mother was not the biological mother of the child; the mother was a co-wife with a selected mother; or either the mother or child was not available for the duration of the study.

### 2.3. Instruments and Protocol

The enumerators administered two structured questionnaires to the respondent. The first questionnaire collected information on household socio-demographics and assets, and factors related to women’s empowerment. The second questionnaire collected information on household mobile phone access and ownership, and perceptions of their experiences with each of the three time allocation data collection methods.

For the criterion time allocation assessment method (i.e., direct observation), enumerators recorded all activities undertaken by the respondent in 15 min intervals (“timeslots”) from approximately 06:00 to 21:00, using a structured instrument comprising 44 activities.

On the day after the observation day, a multiple-pass 24HR was administered to the respondent to collect information on all activities undertaken by the respondent on the previous day. In the first pass, the respondent was asked to list everything she did the previous day; in the second pass, additional details about each activity and any concurrent activities were recorded. The time and duration of each activity were recorded in 15 min increments. In the third pass, the enumerator confirmed with the respondent that each activity was recorded accurately. The 24HR protocol was based on a module developed for the WEAI, which was itself based on the Lesotho Time Budget Study [65,109].

On the observation day, a small, lightweight AWC (iON SnapCam Lite, dimensions 42 × 42 × 13 mm^3^) was attached to a t-shirt worn by the respondent at approximately 06:00 and removed at approximately 21:00. Participants were instructed to wear the AWC while continuing their usual activities, covering or removing the camera as needed for privacy. The AWC automatically recorded a picture every 30 s, storing all photos (approximately 1800) on a memory card.

The following day, an enumerator first reviewed the photos captured by the AWC on a tablet and annotated the activities she thought—based on the photos—were undertaken by the respondent, i.e., the enumerator image interpretation (EII). Based on her interpretation of the photos, the enumerator demarcated the series of activities for review later that day with the respondent. Upon meeting with the respondent, the enumerator first administered the 24HR. The enumerator then administered the IAR by first reviewing the AWC photos with the respondent on the tablet (16GB Samsung Galaxy Tab 3 with a 10” screen, using Simple Gallery software for image display). During this interview, the enumerator used “verbal probing” to elicit from the participant additional relevant information about the activities performed, for example, to elaborate on what she was doing, who she was with, where she was going, and why [110,111]. The enumerator revised her original annotations (i.e., the EII) of activities undertaken by the respondent, as needed, based on the respondent’s feedback.

The IAR protocol was adapted from one described by Kelly et al. (2015). The protocol followed ethical guidelines for AWC research to ensure privacy of the participants was maintained [112]. All protocols were pilot tested and refined prior to the start of the study.

### 2.4. Data Processing

The number of minutes allocated to each of 44 activities recorded over the fifteen-hour period was calculated for each respondent and for each of the 3 data collection methods, in 15 min intervals (“timeslots”). The discrete activities were categorized into the nine mutually exclusive ICATUS-2016 major divisions (“activity groups”): (1) employment and related activities (“employment”), (2) production of goods for own final use (“own production”), (3) unpaid domestic services for household and family members (“domestic chores”), (4) unpaid caregiving services for household and family members (“caregiving”), (5) unpaid volunteer, trainee, and other unpaid work, (6) learning, (7) socializing and communication, community participation, and religious practice (“socializing”), (8) culture, leisure, mass media, and sports practices (“leisure”), and (9) self-care and maintenance (“self-care”), as presented in Supplementary Table S2 [70]. When individuals were observed performing more than one activity concurrently, the activities were given equal weight such that no activity was deemed “primary” or “secondary”.

Of the 44 activities tracked, four were considered to be “simultaneous”, i.e., they could be performed while also performing other activities: care of the index child, care of other children or adults, chatting with friends or family, and watching TV or listening to the radio. When just one activity was performed in a timeslot, the activity performed counted for the entire 15 min. The simultaneous activities were always credited the full 15 min. However, for all other activities, when more than one activity was performed per timeslot, the 15 min were evenly distributed across the activities performed. For example, if in a 15 min timeslot, the participant was snacking (self-care) and then started preparing food (domestic chores) while feeding the index child (caregiving), caregiving—a simultaneous activity—was credited 15 min and self-care and domestic chores were each credited 7.5 min.

The proportion of the study population living below USD 1.25/day was calculated using the Uganda 2012 Poverty Probability Index (PPI) with data collected via the respondents’ questionnaires [113].

### 2.5. Data Analysis

The primary outcome variables analyzed were the total minutes allocated to each of the nine ICATUS-2016 activity groups and the median number of concurrent activities performed across all 15 min timeslots. Data were analyzed using Stata/SE version 15.1. *p*-values less than 0.05 were considered significant for all tests. Cases with incomplete data for any of the three methods (observation, 24HR, or IAR) were eliminated from analysis. Key socio-demographic characteristics for participating and missing households were compared using the Mann–Whitney U two-sample statistic for continuous data, and the Fisher exact test for categorical data.

Due to inter-participant differences in actual observation start and end times and technical challenges with insufficient light in the early morning and evening, the analyses were limited to the 12 h period from 8 a.m. to 8 p.m. to retain as many cases as possible with complete data. The Wilcoxon signed rank sum test was used to compare the distributions of time allocation obtained via the criterion method (observation) versus the 24HR, IAR, or EII. The median time allocated for only those women partaking in each activity was also calculated and compared using the Wilcoxon signed rank sum test. Women’s time allocations estimated via EII and IAR were also calculated for the non-observation day and compared to the corresponding estimates for the observation day using the Wilcoxon signed rank sum test. The Wilcoxon signed rank sum test was also used to compare the distributions of the median number of concurrent activities obtained via the criterion method (observation) versus the 24HR or IAR.

The inter-tool agreement between the criterion method (observation) and 24HR or IAR was assessed using the Bland–Altman limits of agreement (LOA) method for each ICATUS-2016 major division (minutes/d) [106]. Specifically, for each individual, the differences between the methods (the criterion measure of time allocation minus the time allocation estimated using either 24HR or IAR) versus the mean of the two methods were plotted; the bias and the 95% LOA (mean difference ± 2 SD of the differences) were estimated. The numbers of participants for whom the differences between the two methods were greater or less than zero were also calculated. The Bland–Altman LOA approach was used to assess inter-method agreement for estimating the median number of concurrent activities.

Time allocations estimated via the 24HR and IAR methods against the reference method were compared using Cronbach’s (reliability) coefficient alpha. Cronbach’s coefficient alpha was interpreted as follows: <0.70 unacceptable; >0.70 acceptable; >0.80 moderate; 0.90–0.95 high; >0.95 suspect [114]. The inter-method reliability (24HR and IAR methods against the criterion method) for estimating the median number of concurrent activities was compared using the weighted Cohen’s κ coefficient. Cohen’s κ coefficient was interpreted as follows: <0·00 poor agreement; 0·00–0·20 slight agreement; 0·21–0·40 fair agreement; 0·41–0·60 moderate agreement; 0·61–0·80 substantial agreement; 0·81–1·00 almost perfect agreement [115,116].

## 3. Results

### 3.1. Characteristics of the Sample

**Figure 1:**
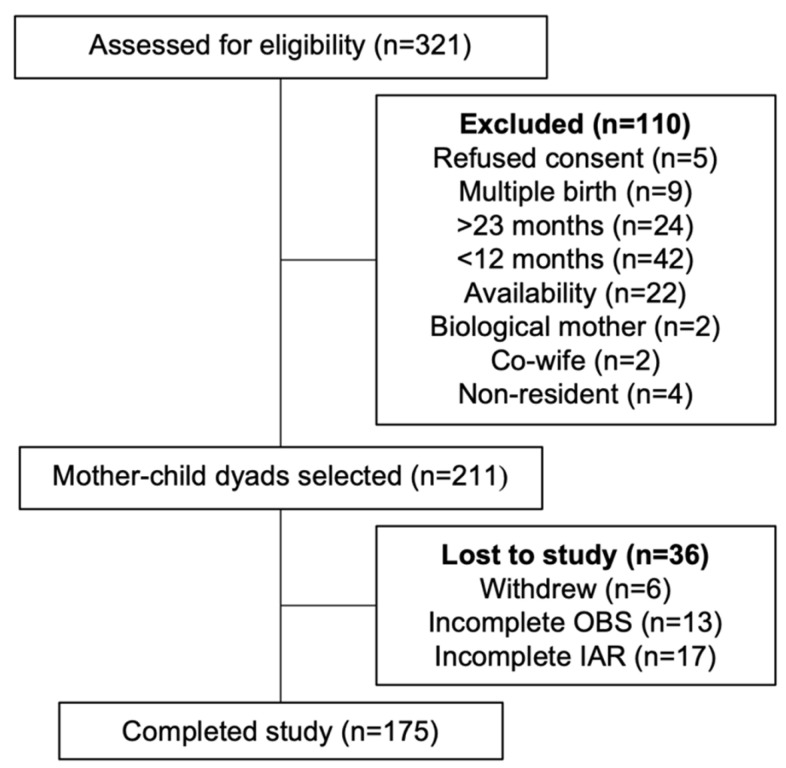
Study population. OBS, observation; IAR, image-assisted recall.

Overall, 211 women were recruited into the study. Among those recruited, six participants voluntarily withdrew, and 30 participants were eliminated from analysis due to incomplete data (Figure 1). Characteristics of the study population are presented and compared with participants who were lost to follow-up or excluded from the analyses in Table 1. These comparisons show some differences between them, including child breastfeeding status (60% for participants vs. 41% for non-participants) and alternative childcare provided exclusively by persons aged thirteen years or older (39% for participants vs. 64% for non-participants). The median household size was six members, and nearly one quarter of participating households lived below USD 1.25/day. Most participating respondents were married and between the ages of 20 and 29 years. Nearly two-thirds of participating respondents had not completed primary school, and just under one half were literate. Most respondents were pregnant, breastfeeding, or both.

The median age of participating children was 17 months, and there were slightly more males (55%) than females (45%). Nearly all children were initially breastfed, although just 60% were breastfeeding at the time of data collection. Among the study participants, over 90% of children were cared for by at least one alternative caregiver (other than their mother), of which more than 60% included at least one alternative caregiver who was less than 13 years of age.

### 3.2. Time Allocation

Most of the work done by the participating women comprised activities traditionally considered to be “reproductive” work rather than “productive” work (Table 2) [117]. For most activity groups, the time allocations were not normally distributed (Supplementary Figure S1a–u). Based on the observation data, women spent over two-thirds of their time (median = 491 min) from 8 a.m. to 8 p.m. providing care. Domestic chores and own production were also important activities for allotted time (median = 318 min and 45 min, respectively). Overall, the highest amount of caregiving time was allotted to care of the index child (median = 405 min) and care of other children or adults (median = 255 min); for domestic chores, the highest amount of time overall was allotted to cooking (median = 85 min) and food preparation (median = 51 min) (Supplementary Table S14). At the population level, there was little time allocated to employment (median = 0 min). Among the women in this study who engaged in employed work (*n* = 77), 16 min (median) were allocated to that activity (Supplementary Table S4). Overall, although women spent about half their time socializing, hardly any time was allocated to other leisure activities (median = 0 min). Women in this study spend much of their time multitasking. The median number of concurrent activities across all timeslots was three (Supplementary Table S5). Women performed more than one activity in 88% of the 48 timeslots (Supplementary Table S6).

The inter-method comparisons show that the median amount of time allocated to caregiving was substantially underestimated by both 24HR (63%) and IAR (15%) (Table 2). Median time allocated to socializing was also substantially underestimated by both the 24HR (52%) and the IAR (30%) methods. Both 24HR and IAR methods accurately estimated the median time allocated to employment and domestic chores. The IAR method accurately estimated the median time allocated to own production whereas that median time was overestimated (9%) by the 24HR. The median time allocated to self-care was underestimated by the 24HR (15%) but overestimated by the IAR (16%). For most activities, median time allocations estimated via the EII (i.e., the enumerator’s interpretation of the wearable camera’s images compiled prior to the IAR) underestimated the observation data, ranging from 7% (domestic chores) to 78% (socializing) (Supplementary Table S3). The median number of concurrent activities was accurately estimated by the IAR but underestimated by the 24HR (Supplementary Table S5). 

Comparing the median number of minutes estimated using the EII or IAR for the observation versus non-observation days showed no significant differences for employment, domestic chores, socializing (IAR only), leisure, and self-care (Supplementary Table S7). However, for both EII and IAR, the median number of minutes allocated to own production on the observation day was lower than the non-observation day (35 min vs. 53 min for EII; 43 min vs. 60 min for IAR), whereas the median number of minutes allocated to caregiving was higher on the observation day than on the non-observation day (315 min vs. 235 min for EII; 418 min vs. 339 min for IAR). For the EII, the median time allocated socializing was also lower on the observation (90 min) than non-observation day (150 min).

### 3.3. Measures of Agreement

The systematic bias differs substantially across activity groups (Table 3). It is low for most activity groups (employment, own production, domestic chores, leisure, and self-care), ranging from 1 min (own production via IAR and domestic chores via 24HR) to 33 min (leisure via 24HR). However, for both methods the bias is high for caregiving (226 min for 24HR and 62 min for IAR) and socializing (172 min for 24HR and 109 min for IAR). For both 24HR and IAR, the percentage of participants with median time allocation estimations that were within 30 min of the criterion method ranged from 5% (caregiving via 24HR) to 79% (employment via 24HR) (Supplementary Table S10). Between 2% (self-care via 24HR) and 79% (caregiving via 24HR) of the time allocation estimates erred by more than two hours. For concurrent activities, there was no systematic bias for IAR whereas 24HR systematically underestimated the median number of concurrent activities by 1.3 (Supplementary Table S12). The difference in the estimated median number of concurrent activities (compared to observation) was less than two for about half (54%) of households via 24HR and three-quarters (74%) via IAR. (Supplementary Table S11). For only 17% and 21% of households (24HR and IAR, respectively), there was no difference in the estimated median number of concurrent activities compared to observation.

**Figure 2:**
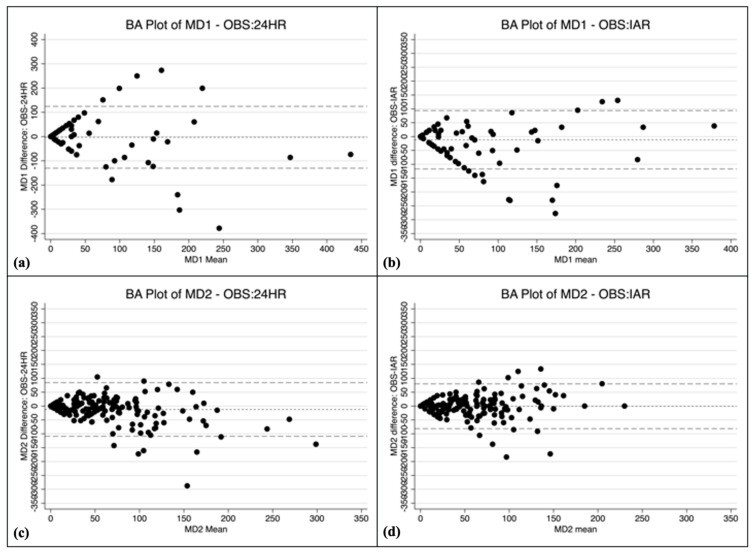

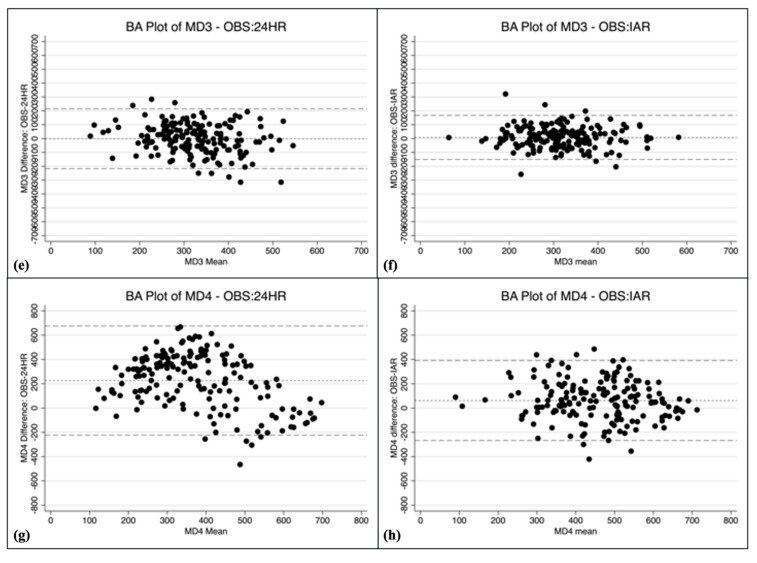

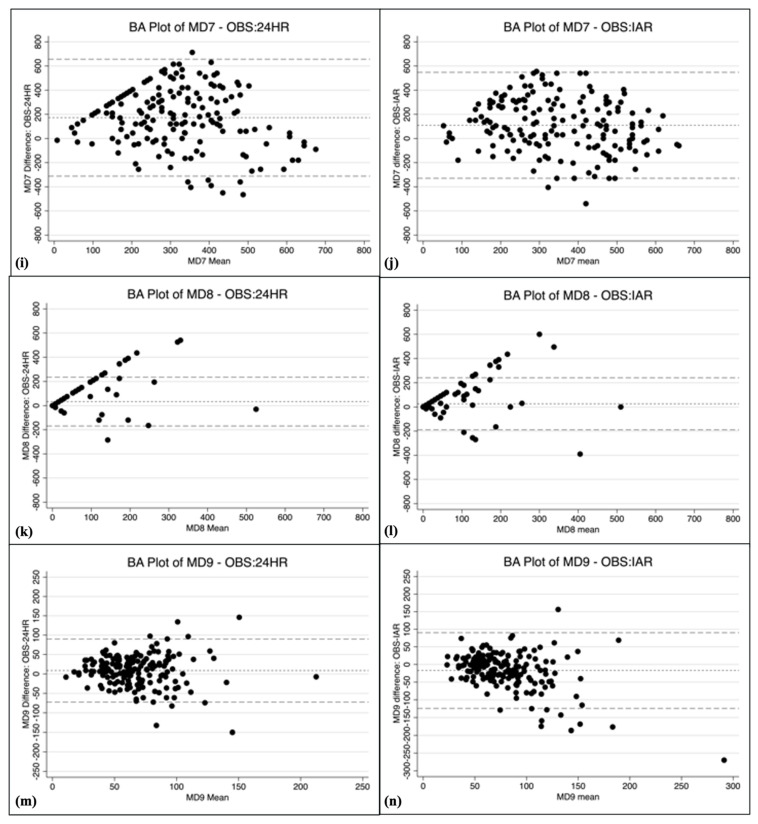
Bland–Altman (BA) plots of time allocation difference versus the mean of observation (OBS) and 24 h recall (24HR) or image assisted recall (IAR). (**a**) BA plot for employment and related activities (MD1), OBS and 24HR, (**b**) BA plot for employment and related activities (MD1), OBS and IAR, (**c**) BA plot for production of goods for own final use (MD2), OBS and 24HR and (**d**) BA plot for production of goods for own final use (MD2), OBS and IAR. (**e**) BA plot for unpaid domestic services for household and family members (MD3), OBS and 24HR, (**f**) BA plot for unpaid domestic services for household and family members (MD3), OBS and IAR, (**g**) BA plot for unpaid caregiving services for household and family members (MD4), OBS and 24HR, and (**h**) BA plot for unpaid caregiving services for household and family members (MD4), OBS and IAR, (**i**) BA plot for socializing and communication, community participation, and religious practice (MD7), OBS and 24HR, (**j**) BA plot for socializing and communication, community participation, and religious practice (MD7), OBS and IAR, (**k**) BA plot for culture, leisure, mass media, and sports practices (MD8), OBS and 24HR, and (**l**) BA plot for culture, leisure, mass media, and sports practices (MD8), OBS and IAR, (**m**) BA plot for self-care and maintenance (MD9), OBS and 24HR, and (**n**) BA plot for self-care and maintenance (MD9), OBS and AR. The dotted line is the mean difference (bias), the long-dashed lines are +/− 2SD limits of agreement (LOA). A bias > 0 indicates that 24HR or IAR underestimates time allocation.

**Figure 3:**
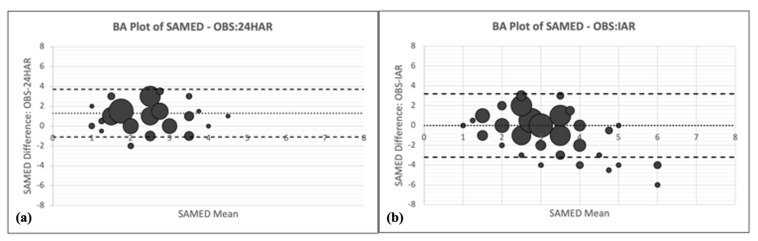
Bland–Altman (BA) plots of time allocation difference versus the mean of observation (OBS) and 24 h recall (24HR) or image-assisted recall (IAR). (**a**) BA plot for concurrent activities-OBS and 24HR, (**b**) BA plot for concurrent activities, OBS and IAR. The dotted line is the mean difference (bias), the long-dashed lines are +/− 2 SD limits of agreement (LOA). A bias > 0 indicates that 24HR or IAR underestimates time allocation. The size of the point corresponds with the number of households.

For 24HR and IAR, the time allocation Bland–Altman plots showed varying patterns across activity groups (Figure 2a–n and Figure 3a,b). Only the IAR method generated cloud-shaped plots (domestic chores, caregiving, and socializing), indicating the method performed equally well for women spending little time doing these activities and women spending substantial time doing these activities. Both the 24HR and the IAR methods had fan-shaped plots for employment, own production (IAR only), and leisure, indicating the amount of random error increased as the mean time allocated to the activity group increased. The 24HR method had several downward-sloping Bland–Altman plots (own production, domestic chores, caregiving, and self-care), and IAR had one downward-sloping plot for self-care, indicating the method underestimated time allocated to the activity for women on the lower end of the spectrum and overestimated time allocated to the activity for women at the upper end of the spectrum. For concurrent activities, the Bland–Altman plot appears cloud shaped for 24HR whereas the plot for IAR appears to be downward sloping.

The width of the LOA varied substantially across activity groups (Table 3). The LOAs were within about 2 h for both methods for employment, own production, self-care, and domestic chores (IAR only). However, the LOAs for caregiving and socializing were high, with overestimates ranging from 223 to 329 min and underestimates ranging from 390 to 675 min. For concurrent activities, the LOA for the 24HR ranged from an overestimate of 1.1 activities to an underestimate of 3.7 activities, and for IAR +/− 3.2 activities (Supplementary Table S12). 

For both the 24HR and IAR methods, Cronbach’s coefficient alpha indicated that the inter-method agreement with observation was unacceptable for most activities (caregiving, socializing, leisure, and self-care) (Table 4). For domestic chores, the reliability was also unacceptable for 24HR but was acceptable for IAR. For own production, the reliability was moderate for 24HR but acceptable for IAR. Reliability for employment for both methods was acceptable. For concurrent activities, Cohen’s κ indicated that agreement was no better than if it had occurred purely by chance (24HR = 0.028; IAR = 0.031) (Supplementary Table S13).

## 4. Discussion

This is the first study to validate the 24HR or IAR methods using an AWC for collecting women’s time-use data in a low-income country context. We assessed the concurrent validity using direct observation as the criterion method with 211 women in the rural Eastern Region of Uganda. The results show the systematic bias for time allocation to employment, own production, domestic chores, and self-care was low, for both the 24HR and IAR, whereas time allocation to caregiving and socializing may be severely underestimated (>1 h) by both methods. The extent of underestimation at the population level was higher for the 24HR than IAR, especially for caregiving (3.5 times higher). This finding is consistent with other studies, which show between a third and three-quarters of respondents’ recall regarding childcare is inaccurate [69], and that, compared with other activities recall errors are highest for caregiving activities such as feeding children, breastfeeding, and supervising children [50].

Several factors may have contributed to the systematic underestimation of time allocation to caregiving and socializing seen in this study. First, most childcare in this study context (rural Uganda) is omnipresent “passive” childcare, that is, constantly performed while simultaneously performing other household chores or chatting, e.g., a mother may supervise a small child at play while washing clothes. Such omnipresent “passive” activities may be so routine as to seem unremarkable to the participant in both the AWC photographic record and in memory [118]. Second, some activities, such as socializing, largely happen “off-camera”, e.g., while washing clothes, the mother may be chatting with a friend who is not in the camera’s field of vision. There is no photographic record of these “background” activities to trigger the participant’s recall. In both examples, the AWC photos used in the IAR may remediate some but not all misreporting which could explain why the LOAs are wider and the systematic bias is higher for the 24HR than IAR. Further support for this interpretation is the numbers of concurrent activities that women performed were higher and accurately estimated by IAR whereas they were underestimated by the 24HR. 

This finding is also consistent with results from previous studies showing, if concurrent activities were taken into account, estimates of women’s time allocation to childcare would increase two-fold [119], and that less than a quarter of time spent on childcare is reported via traditional methods as a “primary” activity [34]. In a multiple-country analysis, estimates of women’s time allocation to childcare increased depending on how concurrent activities were counted, ranging from an increase of 31% (Ethiopia) to 134% (Zimbabwe) [120]. There is a long history of discussion on how to count time allocated to concurrent activities [20,34,43,121]. Most studies avoid dealing with multiple concurrent activities by artificially limiting the number of activities collected or analyzed (e.g., just the “primary” activity). When multiple concurrent activities are allowed, typically the timeslot is equally divided among the concurrent activities, which presumes these activities are performed sequentially [122]. This presumption, however, does not hold true in a rural Uganda setting where childcare or chatting are generally done concurrently with other activities. In this study, if caregiving and socializing had been analyzed in the traditional way (i.e., treating them as sequential rather than simultaneous activities), the estimated median amount of time allocated to these activities would have been reduced by 44% and 66%, respectively (Supplementary Table S15). In research where women’s time use is an outcome of interest, the method of data collection and analysis must account for both concurrent and simultaneous activities to accurately reflect women’s time burdens and social well-being.

For most activities, random error could be high (greater than 2 h), most notably for caregiving and socializing where underestimates could exceed 7 h. Such high LOAs indicate that at the individual level, for most activities, inaccuracies in time estimations can be large using either the 24HR or IAR. If results from 24HR or IAR are used to assess their associations with other variables, attenuation will occur. The finding of large random errors is consistent with other time-use studies, although the cause is unclear [89,123].

For time allocated to domestic chores, caregiving, and socializing, only the IAR showed a Bland–Altman plot pattern having no slope, indicating constant variability of the error. Therefore, compared to 24HR, using the IAR for these activities may result in more predictable bias when using time use as predictor of an outcome variable in regression outcome models. There was a downward slope in the Bland–Altman plots for domestic chores and caregiving (24HR only). Fan-shaped plot patterns were also found (employment, leisure, socializing for 24HR only, and own production for IAR only). Fan-shaped and downward-sloping plot patterns indicate that, for these activities and assessment methods, the magnitude and/or direction of measurement error may change as the amount of time performing the activity increases. This precludes any attempt to predict the consequences of the measurement error on regression outcome models including time use as an exposure/predictor.

Several AWC studies for other outcomes of interest (diet, physical activity, caregiving) did not include an IAR. Instead, a topical expert coded the images based on their interpretation of the activities recorded in the photos, which reduces respondent burden [71,73,75,87,89,95,96,116,124,125,126]. We therefore examined whether the IAR was essential for the interpretation of the AWC photos. In this study, the EII did not provide a reliable estimate of women’s time allocation. The pattern was the same as the IAR but the degree of underestimation compared to direct observation was more severe. For example, the EII underestimated the median number of minutes allocated to caregiving and socializing by 37% and 78%, respectively, compared with 17% and 30%, for the IAR. These results indicate insufficient visual clues were captured for an external coder to determine all activities undertaken in a rural low-income country setting where women primarily work from home.

Several studies have investigated the associations between women’s time allocations and maternal and/or child nutrition-related outcomes [8,11,12,13,14,15,16,17,18,127,128]. The results of these studies, which are based on 24HRs, are often conflicting. The results of this study indicate that previous time-use research relying on the 24HR method, in particular research exploring the associations between women’s time allocations and nutrition outcomes, is likely unreliable.

### Strengths and Limitations

One strength of this study is the use of direct observation as the criterion method. The process of observation, however, might have influenced the participants’ activities or IAR proficiency (e.g., due to heightened awareness of activities performed). Comparisons of time allocation on observation day vs. non-observation day measured via IAR indicate that study participants recalled more time caregiving and less time engaged in own production on observation days than non-observation days. The same is true of the EII, suggesting that the difference in time allocation is real and not just a difference in recall (Supplementary Table S7). More time was spent caregiving on observation days compared to non-observation days regardless of the order in which the household was observed versus administered the IAR method (Supplementary Tables S8 and s9). Even though the increased time spent caregiving might be due to a social desirability or reactivity bias, it may simply reflect a culture of hospitality. The median time spent caregiving remained high on non-observation days (339 min vs. 418 min observed day) and well above that assessed via 24HR on the observation day (180 min), indicating that any changes in activity patterns due to having an observer at home did not substantially contribute to under-reporting of caregiving activities. 

The quality of data captured by the AWC was often compromised by technical issues that have also been reported by previous investigators. These issues include insufficient lighting or poor image quality [75,80,84,85,86,87,89,90,91,92,98,126,129,130,131]; the tedious, time-consuming, and manual processes required to manage and code hundreds of thousands of photos [75,80,88,89,90,98,124]; hardware issues resulting in lost data [71,73,75,76,77,80,81,83,89,90,91,95,98,124,126,131,132,133,134]; and camera-specific software issues (e.g., the built-in filename format by image number rather than timestamp, tendency of the cameras to “lose” time over time). Aside from the functionality of the AWCs, the onerous structure of the IAR protocol for enumerators may have contributed to error. The observation and 24HR protocols were closed-ended and shared a similar matrix structure of pre-specified activity categories, whereas the IAR structure was open-ended to capture a narrative of the activities performed to elicit more detailed information. Some of these difficulties could be remediated in the future with a computer-assisted personal interview (CAPI)-based data collection tool that could prompt enumerators when, for example, a series of recorded activities was not closed.

Although all enumerators and research assistants received the same training in the coding of activities, there were variations in how the same or similar activities were coded between field enumerators and also between IAR data entry research assistants. Inconsistencies in the coding of activities within activity groups posed no problem, since the time allocation was analyzed at the activity group level. For example, playing with a young child and feeding a young child are somewhat ambiguous activities, but both fell under the caregiving activity group. Underestimations in one activity would have offset overestimates in another activity within the same activity group. However, a few activities were coded into different activity groups by field enumerators and data entry research assistants. For example, peeling sweet potatoes and shelling and pounding groundnuts were variably coded as food preparation (domestic chores) or post-harvest processing (own production). To address this issue, post-harvest processing activities were remapped to the domestic chores activity group. While this issue was caught, other coding inconsistencies may have contributed to the high random error seen in this study.

Due, in part, to the poor performance of the AWC in the low-light conditions of early morning and late evening, we restricted the analysis (for all three methods) from the intended 15 h period to a standard 12 h period (8 a.m. to 8 p.m.). Restricting the period of analysis may have influenced the results if one method was better than the other at capturing an activity that occurred primarily outside the 8 a.m. to 8 p.m. period. Upon review (ALSB), however, the only activities that commonly occurred before 8 a.m. or after 8 p.m.—and not any other part of the day—were study-related interactions, which were not included in the analysis. Whereas cooking food and eating often happened after 8 p.m., these activities also always occurred during the day too, so any differences in method performance would be evident. Finally, less than half of the women in this study were engaged in employed work and the data were only collected in one season. The results of the inter-method comparisons may be different in a different season or in a population where a larger proportion of women spent more time in employed work if, for example, one and/or the other method was more effective at measuring time allocated to employment.

## 5. Conclusions

This study aimed to evaluate the concurrent criterion validity, for assessing women’s time use, using an AWC-based IAR method and the 24HR method. Our hypothesis was that prospectively capturing activity data would reduce systematic and random errors inherent to time allocation recalls and reduce respondent/interviewer burdens inherent to observation to allow accurate time allocation data collection at scale for programmatic purposes in rural low-income country contexts. Our results indicate that both the 24HR and IAR provide accurate estimates of the median time allocated to employment, own production (IAR only), and domestic chores at the population level, whereas neither the 24HR nor IAR are valid methods for measuring median time allocated to caregiving or socializing. The high LOAs observed across all activities indicate high random error at the individual level, which will attenuate true associations between time allocation—where estimated via 24HR or IAR—and outcomes of interest. For most activities, neither 24HR nor IAR are valid methods for estimating time allocation at the individual level. To the best of our knowledge, there is no globally accepted threshold LOA for time use, however, a difference of more than two hours (out of twelve hours) seems substantial. The cloud-shaped pattern exhibited only by the IAR-generated Bland–Altman plots for own production, domestic chores, caregiving, and socializing suggest that measurement error due to IAR may be easier to handle and adjust for statistically compared to 24HR when assessing associations of time use with outcome variables for these activities.

This study has important implications for interpreting time-use data collected via 24HR in, for example, the Women’s Empowerment in Agriculture Index (WEAI) standard time-use module [65] or the Living Standards Measurement Study (LSMS) stylized activity log module [135]. It suggests concurrent activities, such as socializing and caregiving, may be under-estimated unless explicitly probed and counted. These results lend credence to modifications made in the time allocation module of the Project-Level Women’s Empowerment in Agriculture Index (Pro-WEAI) to reduce error in measuring caregiving with the addition of checkboxes for each 24HR timeslot indicating whether the participant was caring for a child [136,137]. The same approach may be needed for time spent socializing.

In calculating time allocation estimates for these activities, they should be allotted credit for the entire timeslot duration. Formative research conducted in the study location is also important to understand the activities commonly undertaken by the target population, their purpose, in terms of own use or income generation, and patterns (simultaneous or not) so that they can be properly categorized.

This study shows that, for caregiving, socializing, and domestic chores, the IAR outperforms 24HR. This is important because caregiving and domestic chores are activities most often performed by women. Further work is needed to design an IAR protocol that works in rural low-income country contexts where literacy is low and exposure to first-person perspective photographs is limited. The IAR protocol should be simplified and modified to enable image coding in the field. Instead of reviewing all images with the respondent, it may be more practical and effective to probe the respondent on activities using a few pre-selected (by the enumerator) “sentinel” images per timeslot. Furthermore, enumerator training should include practice recognizing and interpreting problematic activities using AWC photos collected from target populations in the study area, such as breastfeeding and passive caregiving; scanning for contextual clues in individual images and across a series of images; and facilitation skills. Coding consistency across enumerators should be assessed prior to the start of data collection.

Further research is needed to understand how low-literacy populations with limited exposure to first-person perspective photographs cognitively process wearable camera images. The IAR method assumes that AWC-generated photographs will trigger the participants’ memory of activities done on the previous day to improve recall accuracy [100,101,102,103]. If the participants inferred what they were doing from what they saw in the photos rather than used the photos as a memory aid, it would be a very different cognitive task and (possibly) outcome. As far as we are aware, this is the first study to quantify the extent of measurement error, when the 24HR or AWC-IAR are used to estimate women’s time use in a low-income country context. Future research should also assess the magnitude and nature of error in estimating time allocation with 24HR and IAR in other contexts.

## Figures and Tables

**Table 1:** Characteristics of households, mothers, and children participating in and excluded from the analysis.

	Participating				Participants Excluded from Analyses				*p*
	n	%	Median	25th, 75th	n	%	Median	25th, 75th	
Households									
Number of household members			6	5, 8			5	4, 7	0.1017
Living below USD 1.25/day (2005 PPP)	140	24.1			23	21.2			0.086
Mothers									
Age (years)			26	22, 30			23	20, 28	0.0449
15–19	18	10.3			6	16.7			0.521
20–29	105	60			23	63.9			
30–39	44	25.1			6	16.7			
40–49	8	4.6			1	2.8			
Marital status									
Single	19	10.9			2	6.1			0.833
Married or co-habiting	147	84.5			30	90.9			
Level of education									
None or primary incomplete	106	60.1			18	54.6			0.378
Primary complete	62	35.4			12	36.4			
Secondary complete	5	2.9			2	6.1			
Can read and write	82	48			20	60.6			0.127
Maternity status									
Pregnant	25	14.9			8	23.5			0.16
Breastfeeding	110	62.9			17	47.2			0.061
Pregnant or breastfeeding	129	73.7			23	63.9			0.16
Children									
Age (months)			16.7	14.8, 20.0			17.7	14.8, 19.6	0.9001
12–17	104	59.8			19	54.3			0.338
18–23	70	40.2			16	45.7			
Sex									
Female	78	44.6			20	57.1			0.12
Male	97	55.4			15	42.9			
Ever breastfed	172	99.4			31	96.9			0.288
Currently breastfeeding	103	59.5			13	40.6			0.037
Child caregivers			3	2, 4			3	2, 4	0.2597
No alternative caregivers	16	9.1			3	8.3			0.588
All child caregivers > 13 years	68	38.9			23	63.9			0.005

PPP, purchasing power parity; P, *p*-value using Mann–Whitney U test to compare the medians and Fisher’s exact test to compare the categorical data.

**Table 2:** Inter-method comparison of the median time allocated in minutes to activity groups. (Median value, and 25th and 75th percentiles).

ICATUS Activity Group	N	Non-Participation	OBS		24HR		IAR	
		**n (%)**	**Median** **(min)**	**25th, 75th**	**Median** **(min)**	**25th, 75th**	**Median** **(min)**	**25th, 75th**
Employment and related activities (MD1)	175	98 (56.0)	0	0, 5	0	0, 0	0	0, 35
Production of goods for own final use (MD2)	175	16 (9.1)	45	10, 79	49 †	15, 90	43	18, 81
Unpaid domestic services for household and family members (MD3)	175	0 (0.0)	318	263, 370	320	245, 396	311	251, 374
Unpaid caregiving services for household and family member (MD4) *	175	0 (0.0)	491	388, 608	180 †	96, 390	418 †	324, 541
Socializing and communication, community participation and religious practice (MD7) *	175	0 (0.0)	405	270, 525	195 †	75, 330	285 †	105, 465
Culture, leisure, mass media and sports practices (MD8) *	175	102 (58.3)	0	0, 30	0 †	0, 0	0 †	0, 0
Self-care and maintenance (MD9)	175	0 (0.0)	68	50, 88	58 †	39, 80	79 †	53, 111

OBS, observation; 24HR, 24 h recall; IAR, image-assisted recall. * Activity group contains one or more simultaneous activities. † *p*-value of Wilcoxon signed rank sum test of median time allocated compared to OBS < 0.05. NB: There were no activities that mapped to ICATUS MD5, unpaid volunteer, training, or other unpaid work. NB: This table does not include resting or sleeping (due to known inconsistencies with recording) or answering the phone for the study or other interactions for the study.

**Table 3:** Inter-method comparison of the time allocation bias and limits of agreement (LOA).

	Bias † (min)	LOA ‡ min (h)	
**Employment and related activities (MD1)**			
24HR	−3	−130 (−2)	124 (2)
IAR	−12	−117 (−2)	94 (2)
**Production of goods for own final use (MD2)**			
24HR	−12	−109 (−2)	84 (1)
IAR	−1	−81 (−1)	80 (1)
**Unpaid domestic services for household and family members (MD3)**			
24HR	−1	−217 (−4)	215 (3)
IAR	8	−151 (−2)	167 (3)
Unpaid caregiving services for household and family members (MD4) *			
24HR	226	−223 (−4)	675 (11)
IAR	62	−267 (−4)	390 (7)
Socializing and communication, community participation, and religious practice (MD7) *			
24HR	172	−312 (−5)	656 (11)
IAR	109	−329 (−5)	548 (9)
Culture, leisure, mass media, and sports practices (MD8) *			
24HR	33	−169 (−3)	236 (4)
IAR	26	−189 (−3)	241 (4)
**Self-care and maintenance (MD9)**			
24HR	9	−73 (−1)	90 (2)
IAR	−17	−124 (−2)	90 (2)

LOA, limits of agreement; 24HR, 24 h recall; IAR, image-assisted recall. * Activity group contains one or more simultaneous activities. † Mean difference. ‡ +/− 2 SD from the mean difference. NB: A negative indicates that 24HR/IAR overestimated OBS.

**Table 4:** Inter-method comparison of reliability for time allocation.

ICATUS Activity Group	24HR		IAR	
	alpha	Score †	alpha	Score †
Employment and related activities (MD1)	0.7347	acceptable	0.7847	acceptable
Production of goods for own final use (MD2)	0.8056	moderate	0.7938	acceptable
Unpaid domestic services for household and family members (MD3)	0.6014	unacceptable	0.7618	acceptable
Unpaid caregiving services for household and family members (MD4) *	0.2901	unacceptable	0.4273	unacceptable
Socializing and communication, community participation, and religious practice (MD7) *	0.1728	unacceptable	0.2270	unacceptable
Culture, leisure, mass media, and sports practices (MD8) *	0.5107	unacceptable	0.3881	unacceptable
Self-care and maintenance (MD9)	0.4455	unacceptable	0.3792	unacceptable

24HR, 24 h recall; IAR, image-assisted recall; CI, confidence interval. * Activity group contains one or more simultaneous activities. † Using Cronbach’s (reliability) coefficient alpha. Nunnally (1978) and Peterson (1994) suggest the following benchmark scale for interpreting the alpha statistic: <0.70 unacceptable; >0.70 acceptable; >0.80 moderate; 0.90–0.95 high; >0.95 suspect.

## Data Availability

The data that support the findings of this study are openly available in Dataverse at (URL), reference number (reference number).

## Supplementary Materials

The following supporting information can be downloaded at: https://www.mdpi.com/article/10.3390/nu14091833/s1, Figure S1: Plots of the quantiles of activities against the quantiles of the normal distribution (Q–Q plot); Table S1: Household data collection patterns; Table S2: Time-use activities and ICATUS major divisions; Table S2: Time-use activities and ICATUS major divisions; Table S3: Inter-method comparison of the median time allocated in minutes to activity groups, including enumerator image interpretation (EII); Table S4: Inter-method comparison of the median time allocated in minutes to activity groups for participating women only; Table S5: Inter-method comparison of the median number of concurrent activities; Table S6: Inter-method comparison of the median number and proportion of timeslots containing concurrent activities; Table S7: Inter-method comparison of the median time allocated in minutes to activity groups, observation day versus non-observation day; Table S8: Inter-method comparison of the median time allocated in minutes to activity groups, observation day versus non-observation day for households having IAR administered before OBS only; Table S9: Inter-method comparison of the median time allocated in minutes to activity groups, observation day versus non-observation day for households having IAR administered after OBS only; Table S10: Frequency of inter-method time allocation differences; Table S11: Frequency of inter-method median concurrent activities differences; Table S12: Inter-method comparison of the median concurrent activities bias and limits of agreement (LOA); Table S13: Inter-method comparison of reliability for median concurrent activities; Table S12: Inter-method comparison of the median time allocated in minutes to discrete activities; Table S15: Within-method comparison of time allocation—with and without simultaneous activities.

## References

[1] Engle P.L., Menon P., Haddad L. (1997). Care and Nutrition: Concepts and Measurement.

[2] Blackden C.M., Wodon Q. (2006). Gender, Time Use, and Poverty in Sub-Saharan Africa.

[3] OECD (2019). Enabling Women’s Economic Empowerment: New Approaches to Unpaid Care Work in Developing Countries.

[4] International Labour Office (2016). Women at Work: Trends 2016.

[5] Verick S. (2014). Female labor force participation in developing countries. IZA World Labor.

[6] Marini M., Shelton B. (1993). Measuring Household Work: Recent Experience in the United States. Soc. Sci. Res..

[7] Kadiyala S., Harris J., Headey D., Yosef S., Gillespie S. (2014). Agriculture and nutrition in India: Mapping evidence to pathways. Ann. N. Y. Acad. Sci..

[8] Komatsu H., Malapit H.J.L., Theis S. (2018). Does women’s time in domestic work and agriculture affect women’s and children’s dietary diversity? Evidence from Bangladesh, Nepal, Cambodia, Ghana, and Mozambique. Food Policy.

[9] Molyneux M. (2006). Mothers at the Service of the New Poverty Agenda: Progresa/Oportunidades, Mexico’s Conditional Transfer Programme. Soc. Policy Adm..

[10] Senauer B. (1988). The Impact of the Value of Women’s Time on Food and Nutrition in Developing Countries.

[11] Jones A.D., Agudo Y.C., Galway L., Bentley J., Pinstrup-Andersen P. (2012). Heavy agricultural workloads and low crop diversity are strong barriers to improving child feeding practices in the Bolivian Andes. Soc. Sci. Med..

[12] Van den Bold M., Bliznashka L., Ramani G., Olney D., Quisumbing A., Pedehombga A., Ouedraogo M. (2021). Nutrition-sensitive agriculture programme impacts on time use and associations with nutrition outcomes. Matern. Child Nutr..

[13] Ricci J.A., Jerome N.W., Sirageldin I., Aly H., Moussa W., Galal O., Harrison G.G., Kirksey A. (1996). The significance of children’s age in estimating the effect of maternal time use on children’s well-being. Soc. Sci. Med..

[14] Popkin B.M. (1980). Time allocation of the mother and child nutrition. Ecol. Food Nutr..

[15] Gryboski K.L. (1996). Maternal and Non-maternal Time Allocation to Infant Care, and Care during Infant Illness in Rural Java, Indonesia. Soc. Sci. Med..

[16] Hawkes K., O’Connell J.F., Jones N.G.B. (1997). Hadza Women’s Time Allocation, Offspring Provisioning, and the Evolution of Long Postmenopausal Life Spans. Curr. Anthr..

[17] Nordang S., Shoo T., Holmboe-Ottesen G., Kinabo J., Wandel M. (2015). Women’s work in farming, child feeding practices and nutritional status among under-five children in rural Rukwa, Tanzania. Br. J. Nutr..

[18] Bamji M.S., Thimayamma B. (2000). Impact of women’s work on maternal and child nutrition. Ecol. Food Nutr..

[19] Hirway I., Jose S. (2011). Understanding Women’s Work Using Time-Use Statistics: The Case of India. Fem. Econ..

[20] Fedick C.B., Pacholok S., Gauthier A.H. (2005). Methodological issues in the estimation of parental time–Analysis of measures in a Canadian time-use survey. Electron. Int. J. Time Use Res..

[21] Buvinić M., King E.M. Invisible No More? A Methodology and Policy Review of How Time Use Surveys Measure Unpaid Work. United Nations Foundation, Data2X. https://data2x.org/resource-center/invisible-no-more-a-methodology-and-policy-review-of-how-time-use-surveys-measure-unpaid-work-volume-1/.

[22] Bério A.-J. (1984). The analysis of time allocation and activity patterns in nutrition and rural development planning. Food Nutr. Bull..

[23] Walthery P., Gershuny J. (2019). Improving Stylised Working Time Estimates with Time Diary Data: A Multi Study Assessment for the UK. Soc. Indic. Res..

[24] Kanwar P., Yadav D., Sharma N. (2003). Time use pattern of hill farm women: A study in Himachal Pradesh. Himachal. J. Agric. Res..

[25] Szalai A. (1975). Women’s time: Women in the light of contemporary time-budget research. Futures.

[26] Engle P., Lhotská L., Armstrong H. (1997). The Care Initiative: Assessment, Analysis and Action to Improve Care for Nutrition.

[27] Floro M.S., King E.M. (2016). The present and future of time-use analysis in developing countries. Asia-Pac. Popul. J..

[28] Johnston D., Stevano S., Malapit H.J.L., Hull E., Kadiyala S. (2015). Agriculture, Gendered Time Use, and Nutritional Outcomes: A Systematic Review.

[29] Mcguire J.S., Popkin B.M. (1990). Helping Women Improve Nutrition in the Developing World.

[30] Floro M.S. (1995). Economic restructuring, gender and the allocation of time. World Dev..

[31] Nti C.A., Inkumsah D., Fleischer G. (1999). Influence of women’s workload on their nutritional status in selected communities in Ghana. J. Consum. Stud. Home Econ..

[32] Leslie J. (1989). Women’s time: A factor in the use of child survival technologies?. Health Policy Plan.

[33] Leslie J., Paolisso M.J. (1989). Women, Work, and Child Welfare in the Third World.

[34] Ironmonger D. There are only 24 Hours in a Day! Solving the problematic of simultaneous time. Proceedings of the 25th IATUR Conference on Time Use Research.

[35] Irani L., Vemireddy V. (2020). Getting the measurement right! Quantifying time poverty and multitasking from childcare among mothers with children across different age groups in rural north India. Asian Popul. Stud..

[36] Peterman A., Ng S.W., Palermo T., Lee I.-H.E. (2013). Managing the Double Burden: Pregnancy and Labor-Intensive Time Use in Rural China, Mexico, and Tanzania. Stud. Fam. Plan..

[37] Mattingly M.J., Blanchi S.M. (2003). Gender Differences in the Quantity and Quality of Free Time: The, U.S. Experience. Soc. Forces.

[38] Zick C.D., Bryant W. (1996). A New Look at Parents’ Time Spent in Child Care: Primary and Secondary Time Use. Soc. Sci. Res..

[39] Floro M.S. (1995). Women’s well-being, poverty, and work intensity. Fem. Econ..

[40] Ilahi N. (2000). The Intra-household Allocation of Time and Tasks: What Have We Learnt from the Empirical Literature? Policy Research Report on Gender and Development Working Paper.

[41] Acharya M., Bennett L. (1982). Women and the Subsistence Sector: Economic Participation and Household Decisionmaking in Nepal.

[42] As D. (1978). Studies of Time-Use: Problems and Prospects. Acta Sociol..

[43] Budlender D. (2007). A Critical Review of Selected Time Use Surveys.

[44] Budlender D. (2008). The Statistical Evidence on Care and Non-Care Work across Six Countries.

[45] ILO Department of Statistics (2018). Survey Methods to Improve Measurement of Paid and Unpaid Work: Country Practices in Time-Use Measurement.

[46] Andorka R. (1987). Time budgets and their uses. Annu. Rev. Sociol..

[47] European Union (2019). Harmonised European Time Use Surveys (HETUS) 2018 Guidelines.

[48] Michelson W. (1975). Time Budgets and Social Activity. Meeting of the Working Group on Time-Budgets and Social Activity.

[49] Hirway I. (2010). Time-Use Surveys in Developing Countries: An Assessment. Unpaid Work and the Economy.

[50] Ricci J., Jerome N., Megally N., Galal O., Harrison G., Kirksey A. (1995). Assessing the Validity of Informant Recall: Results of a Time Use Pilot Study in Peri-Urban Egypt. Hum. Organ..

[51] Robinson J.P., Pentland W.E., Harvey A.S., Lawton M.P., McColl M.A. (1999). The time-diary method: Structure and uses. Time Use in the Social Sciences.

[52] Gross D.R. (1984). Time allocation: A tool for the study of cultural behavior. Annu. Rev. Anthropol..

[53] Kes A., Swaminathan H., Blackden C.M., Wodon Q. (2006). Gender and Time Poverty in Sub-Saharan Africa. Time Use, and Poverty in Sub-Saharan Africa.

[54] Kalton G., Juster F.T., Stafford F.P. (1985). Sample Design Issues in Time Diary Studies. Time, Goods, and Well-Being.

[55] Robinson J.P., Juster F.T., Stafford F.P. (1985). The Validity and Reliability of Diaries Versus Alternative Time Use Measures. Time, Goods, and Well-Being.

[56] Wagenbuur H.T.M. (1972). An Analysis of the Time-Budget and of the Production and Productivity of Lime Farmers in Southern Ghana.

[57] Gardner F. (2000). Methodological Issues in the Direct Observation of Parent–Child Interaction: Do Observational Findings Reflect the Natural Behavior of Participants?. Clin. Child Fam. Psychol. Rev..

[58] Repp A.C., Nieminen G.S., Olinger E., Brusca R. (1988). Direct Observation: Factors Affecting the Accuracy of Observers. Counc. Rev..

[59] Richer J. (2017). Direct Observation: Impediments and Approaches. Hum. Ethol..

[60] Seymour G., Malapit H., Quisumbing A. (2016). Measuring Time Use in Development Settings.

[61] Juster F.T., Ono H., Stafford F.P. (2003). An Assessment of Alternative Measures of Time Use. Sociol. Methodol..

[62] Brunnich B., Druce P., Ghissassi M., Johnson M., Majidi N., Radas A.L., Riccheri P.R., Camille de Sentenac C., Vacarr D. (2005). Three Case Studies of Time Use Survey Application in Lower and Middle-Income Countries.

[63] Belli R.F., Schwarz N., Singer E., Talarico J. (2000). Decomposition can harm the accuracy of behavioural frequency reports. Appl. Cogn. Psychol..

[64] Seymour G., Malapit H., Quisumbing A. (2020). Measuring Time Use in Developing Country Agriculture: Evidence from Bangladesh and Uganda. Fem. Econ..

[65] Alkire S., Meinzen-Dick R., Peterman A., Quisumbing A.R., Seymour G. (2013). The Women’s Empowerment in Agriculture Index.

[66] Rubin D.C., Baddeley A.D. (1989). Telescoping is not time compression: A model. Mem. Cogn..

[67] Tourangeau R., Jabine T., Straf M., Tanur J., Tourangeau R. (1984). Cognitive Sciences and Survey Methods. Cognitive Aspects of Survey Methodology: Building a Bridge between Disciplines.

[68] Jacobs J.A. (1998). Measuring time at work: Are self-reports accurate?. Mon. Lab. Rev..

[69] Bernard H.R., Killworth P., Kronenfeld D., Sailer L. (1984). The Problem of Informant Accuracy: The Validity of Retrospective Data. Annu. Rev. Anthropol..

[70] United Nations Statistics Division (2019). International Classification of Activities for Time-Use Statistics 2016 (ICATUS 2016).

[71] Arab L., Winter A. (2010). Automated Camera-Phone Experience with the Frequency of Imaging Necessary to Capture Diet. J. Am. Diet. Assoc..

[72] Arab L., Estrin D., Kim D.H., Burke J., Goldman J. (2011). Feasibility testing of an automated image-capture method to aid dietary recall. Eur. J. Clin. Nutr..

[73] Beltran A., Dadabhoy H., Ryan C., Dholakia R., Jia W., Baranowski J., Sun M., Baranowski T. (2018). Dietary Assessment with a Wearable Camera among Children: Feasibility and Intercoder Reliability. J. Acad. Nutr. Diet..

[74] Kamar M., Evans C., Hugh-Jones S. (2019). Factors Influencing British Adolescents’ Intake of Whole Grains: A Pilot Feasibility Study Using SenseCam Assisted Interviews. Nutrients.

[75] Chan V., Davies A., Wellard-Cole L., Lu S., Ng H., Tsoi L., Tiscia A., Signal L., Rangan A., Gemming L. (2021). Using Wearable Cameras to Assess Foods and Beverages Omitted in 24 Hour Dietary Recalls and a Text Entry Food Record App. Nutrients.

[76] Gemming L., Doherty A., Kelly P., Utter J., Ni Mhurchu C. (2013). Feasibility of a SenseCam-assisted 24-h recall to reduce under-reporting of energy intake. Eur. J. Clin. Nutr..

[77] O’Loughlin G., Cullen S.J., McGoldrick A., Connor S., Blain R., O’Malley S., Warrington G. (2013). Using a Wearable Camera to Increase the Accuracy of Dietary Analysis. Am. J. Prev. Med..

[78] Jia W., Chen H.-C., Yue Y., Li Z., Fernstrom J., Bai Y., Li C., Sun M. (2013). Accuracy of food portion size estimation from digital pictures acquired by a chest-worn camera. Public Health Nutr..

[79] Gemming L. (2015). Image-Assisted Dietary Assessment: Evaluating the Potential of Wearable Cameras to Enhance Self-Report in the 24-Hour Dietary Recall Method.

[80] Gemming L., Doherty A., Utter J., Shields E., Ni Mhurchu C. (2015). The use of a wearable camera to capture and categorise the environmental and social context of self-identified eating episodes. Appetite.

[81] Gemming L., Rush E., Maddison R., Doherty A.R., Gant N., Utter J., Ni Mhurchu C. (2014). Wearable cameras can reduce dietary under-reporting: Doubly labelled water validation of a camera-assisted 24 h recall. Br. J. Nutr..

[82] Gemming L., Ni Mhurchu C. (2016). Dietary under-reporting: What foods and which meals are typically under-reported?. Eur. J. Clin. Nutr..

[83] Pettitt C., Liu J., Kwasnicki R.M., Yang G.-Z., Preston T., Frost G. (2015). A pilot study to determine whether using a lightweight, wearable micro-camera improves dietary assessment accuracy and offers information on macronutrients and eating rate. Br. J. Nutr..

[84] Kelly P., Doherty A., Berry E., Hodges S., Batterham A.M., Foster C. (2011). Can we use digital life-log images to investigate active and sedentary travel behaviour? Results from a pilot study. Int. J. Behav. Nutr. Phys. Act..

[85] Kelly P., Doherty A.R., Hamilton A., Matthews A., Batterham A.M., Nelson M., Foster C., Cowburn G. (2012). Evaluating the Feasibility of Measuring Travel to School Using a Wearable Camera. Am. J. Prev. Med..

[86] Kelly P. (2013). Assessing the Utility of Wearable Cameras in the Measurement of Walking and Cycling. Ph.D. Thesis.

[87] Kerr J., Marshall S.J., Godbole S., Chen J., Legge A., Doherty A.R., Kelly P., Oliver M., Badland H.M., Foster C. (2013). Using the SenseCam to Improve Classifications of Sedentary Behavior in Free-Living Settings. Am. J. Prev. Med..

[88] Oliver M., Doherty A.R., Kelly P., Badland H.M., Mavoa S., Shepherd J., Kerr J., Marshall S., Hamilton A., Foster C. (2013). Utility of passive photography to objectively audit built environment features of active transport journeys: An observational study. Int. J. Health Geogr..

[89] Kelly P., Doherty A., Mizdrak A., Marshall S., Kerr J., Legge A., Godbole S., Badland H., Oliver M., Foster C. (2014). High group level validity but high random error of a self-report travel diary, as assessed by wearable cameras. J. Transp. Health.

[90] Barr M., Signal L., Jenkin G., Smith M. (2014). Capturing exposures: Using automated cameras to document environmental determinants of obesity. Health Promot. Int..

[91] Cowburn G., Matthews A., Doherty A., Hamilton A., Kelly P., Williams J., Foster C., Nelson M. (2015). Exploring the opportunities for food and drink purchasing and consumption by teenagers during their journeys between home and school: A feasibility study using a novel method. Public Health Nutr..

[92] Signal L., Stanley J., Smith M., Barr M., Chambers T., Zhou J., Duane A., Gurrin C., Smeaton A., McKerchar C. (2017). Children’s everyday exposure to food marketing: An objective analysis using wearable cameras. Int. J. Behav. Nutr. Phys. Act..

[93] Liu W., Barr M., Pearson A.L., Chambers T., Pfeiffer K., Smith M., Signal L. (2019). Space-time analysis of unhealthy food advertising: New Zealand children’s exposure and health policy options. Health Promot. Int..

[94] McKerchar C., Smith M., Stanley J., Barr M., Chambers T., Abel G., Lacey C., Gage R., Ni Mhurchu C., Signal L. (2019). Food store environment examination–FoodSee: A new method to study the food store environment using wearable cameras. Glob. Health Promot..

[95] Smith M., Stanley J., Signal L., Barr M., Chambers T., Balina A., Mhurchu C.N., Wilson N. (2019). Children’s healthy and unhealthy beverage availability, purchase and consumption: A wearable camera study. Appetite.

[96] Veatupu L., Puloka V., Smith M., McKerchar C., Signal L. (2019). Me’akai in Tonga: Exploring the Nature and Context of the Food Tongan Children Eat in Ha’apai Using Wearable Cameras. Int. J. Environ. Res. Public Health.

[97] Zhou Q., Wang D., Ni Mhurchu C., Gurrin C., Zhou J., Cheng Y., Wang H. (2019). The use of wearable cameras in assessing children’s dietary intake and behaviours in China. Appetite.

[98] Bulungu A.L.S., Palla L., Priebe J., Forsythe L., Katic P., Varley G., Galinda B.D., Sarah N., Nambooze J., Wellard K. (2020). Validation of a life-logging wearable camera method and the 24-h diet recall method for assessing maternal and child dietary diversity. Br. J. Nutr..

[99] Kohrt B., Rai S., Vilakazi K., Thapa K., Bhardwaj A., Van Heerden A. (2019). Procedures to Select Digital Sensing Technologies for Passive Data Collection With Children and Their Caregivers: Qualitative Cultural Assessment in South Africa and Nepal. JMIR Pediatr. Parent..

[100] Chow T.E., Rissman J. (2017). Neurocognitive mechanisms of real-world autobiographical memory retrieval: Insights from studies using wearable camera technology. Ann. N. Y. Acad. Sci..

[101] Hodges S., Williams L., Berry E., Izadi S., Srinivasan J., Butler A., Smyth G., Kapur N., Wood K. SenseCam: A Retrospective Memory Aid. Proceedings of the International conference on ubiquitous computing.

[102] Kelly P., Thomas E., Doherty A., Harms T., Burke Ó., Gershuny J., Foster C. (2015). Developing a Method to Test the Validity of 24 Hour Time Use Diaries Using Wearable Cameras: A Feasibility Pilot. PLoS ONE.

[103] Small L., Sidora-Arcoleo K., Vaughan L., Creed-Capsel J., Chung K.-Y., Stevens C. (2009). Validity and Reliability of Photographic Diet Diaries for Assessing Dietary Intake Among Young Children. ICAN Infant Child Adolesc. Nutr..

[104] Gershuny J., Harms T., Doherty A., Thomas E., Milton K., Kelly P., Foster C. (2017). CAPTURE24: Testing Self-Report Time-Use Diaries Against Objective Instruments in Real Time.

[105] Harms T., Gershuny J., Doherty A., Thomas E., Milton K., Foster C. (2019). A validation study of the Eurostat harmonised European time use study (HETUS) diary using wearable technology. BMC Public Health.

[106] Bland J.M., Altman D.G. (1986). Statistical Methods for Assessing Agreement Between Two Methods of Clinical Measurement. Lancet.

[107] Giavarina D. (2015). Understanding Bland Altman analysis. Biochem. Med..

[108] De Vet H.C.W., Terwee C.B., Mokkink L.B., Knol D.L. (2011). Measurement in Medicine: A Practical Guide.

[109] Lesotho B.S. (2003). Household Budget Survey Report, Lesotho.

[110] Willis G.B. (1999). Cognitive Interviewing: A “How To” Guide.

[111] Ericsson K.A., Simon H.A. (1980). Verbal reports as data. Psychol. Rev..

[112] Kelly P., Marshall S.J., Badland H., Kerr J., Oliver M., Doherty A.R., Foster C. (2013). An Ethical Framework for Automated, Wearable Cameras in Health Behavior Research. Am. J. Prev. Med..

[113] Schreiner M. (2016). Uganda 2012 Poverty Probability Index (PPI).

[114] Peterson R.A. (1994). A Meta-Analysis of Cronbach’s Coefficient Alpha. J. Consum. Res..

[115] Landis J.R., Koch G.G. (1977). The Measurement of Observer Agreement for Categorical Data. Biometrics.

[116] Farooq M., Doulah A., Parton J., McCrory M.A., Higgins J.A., Sazonov E. (2019). Validation of Sensor-Based Food Intake Detection by Multicamera Video Observation in an Unconstrained Environment. Nutrients.

[117] White B. (1984). Measuring time allocation, decision-making and agrarian changes affecting rural women: Examples from recent research in Indonesia. IDS Bull..

[118] Wilson G., Aitken D., Hodgson P., Bailey C. (2019). The hidden impact of home adaptations: Using a wearable camera to explore lived experiences and taken-for-granted behaviours. Health Soc. Care Community.

[119] Floro M.S., Miles M. (2003). Time use, work and overlapping activities: Evidence from Australia. Camb. J. Econ..

[120] Rost L., Bates K., Dellepiane L. (2015). Women’s Economic Empowerment and Care: Evidence for Influencing.

[121] Mueller C. (2018). Time Use Data: Sources and Applications of Data on Paid and Unpaid Labour.

[122] Lentz E., Kerr R.B., Patel R., Dakishoni L., Lupafya E. (2018). The Invisible Hand that Rocks the Cradle: On the Limits of Time Use Surveys. Dev. Chang..

[123] Kan M.Y., Pudney S. (2008). Measurement Error in Stylized and Diary Data on Time Use. Sociol. Methodol..

[124] Raber M., Patterson M., Jia W., Sun M., Baranowski T. (2018). Utility of eButton images for identifying food preparation behaviors and meal-related tasks in adolescents. Nutr. J..

[125] Lee R., Skinner A., Bornstein M.H., Radford A.N., Campbell A., Graham K., Pearson R.M. (2017). Through babies’ eyes: Practical and theoretical considerations of using wearable technology to measure parent–infant behaviour from the mothers’ and infants’ view points. Infant. Behav. Dev..

[126] Raber M., Baranowski T., Crawford K., Sharma S.V., Schick V., Markham C., Jia W., Sun M., Steinman E., Chandra J. (2020). The Healthy Cooking Index: Nutrition Optimizing Home Food Preparation Practices across Multiple Data Collection Methods. J. Acad. Nutr. Diet..

[127] Tucker K., Sanjur D. (1988). Maternal employment and child nutrition in Panama. Soc. Sci. Med..

[128] Wandel M., Holmboe-Ottesen G. (1992). Women’s Work in Agriculture and Child Nutrition in Tanzania. J. Trop. Pediatr..

[129] Laskaris Z., Milando C., Batterman S., Mukherjee B., Basu N., O’Neill M.S., Robins T.G., Fobil J.N. (2019). Derivation of Time-Activity Data Using Wearable Cameras and Measures of Personal Inhalation Exposure among Workers at an Informal Electronic-Waste Recovery Site in Ghana. Ann. Work Expo. Health.

[130] Alharbi R., Stump T., Vafaie N., Pfammatter A., Spring B., Alshurafa N. (2018). I Can’t Be Myself. Proc. ACM Interact. Mob. Wearable Ubiquitous Technol..

[131] Chambers T., Pearson A., Kawachi I., Rzotkiewicz Z., Stanley J., Smith M., Barr M., Ni Mhurchu C., Signal L. (2017). Kids in space: Measuring children’s residential neighborhoods and other destinations using activity space GPS and wearable camera data. Soc. Sci. Med..

[132] Gelonch O., Ribera M., Codern-Bové N., Ramos S., Quintana M., Chico G., Cerulla N., Lafarga P., Radeva P., Garolera M. (2019). Acceptability of a lifelogging wearable camera in older adults with mild cognitive impairment: A mixed-method study. BMC Geriatr..

[133] Sellen A.J., Fogg A., Aitken M., Hodges S., Rother C., Wood K. Do Life-Logging Technologies Support Memory for the Past?. Proceedings of the SIGCHI Conference on Human Factors in Computing Systems.

[134] Wilson G., Jones D., Schofield P., Martin D.J. (2016). Experiences of using a wearable camera to record activity, participation and health-related behaviours: Qualitative reflections of using the Sensecam. Digit. Health.

[135] Grosh M., Glewwe P. (2000). Designing Household Survey Questionnaires for Developing Countries: Lessons from 15 Years of the Living Standards Measurement Study.

[136] Malapit H., Quisumbing A., Meinzen-Dick R., Seymour G., Martinez E.M., Heckert J., Rubin D., Vaz A., Yount K.M. (2019). Development of the project-level Women’s Empowerment in Agriculture Index (pro-WEAI). World Dev..

[137] International Food Policy Research Institute (2020). Project-Level Women’s Empowerment in Agriculture Index (Pro-WEAI) Pilot Version.

