# Supplementary material for: Validation of an Automated Wearable Camera-Based Image-Assisted Recall Method and the 24-h Recall Method for Assessing Women’s Time Allocation in a Nutritionally Vulnerable Population: The Case of Rural Uganda
Source: Nutrients. 2022 Apr 27;14(9):1833. doi: 10.3390/nu14091833 (PMC9101468; doi:10.3390/nu14091833)
Supplement: Supplementary file 1 [file nutrients-14-01833-s001.zip › nutrients-1685033-supplementary.pdf]

SUPPLEMENTARY TABLES AND FIGURES

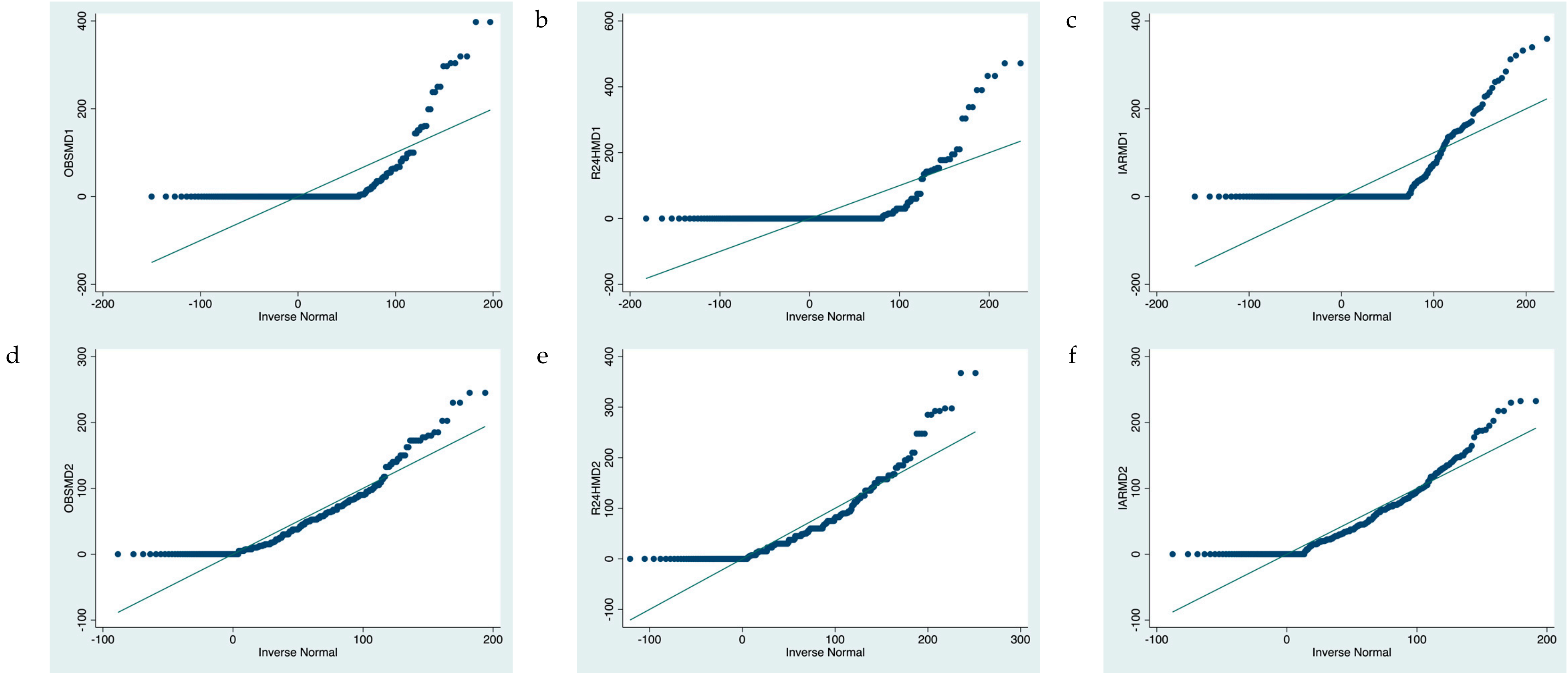

g

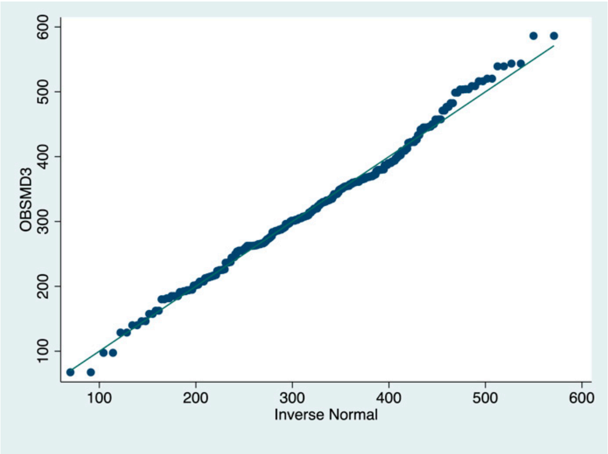
OBSMD3
100 200 300 400 500 600
Inverse Normal
100 200 300 400 500 600

h

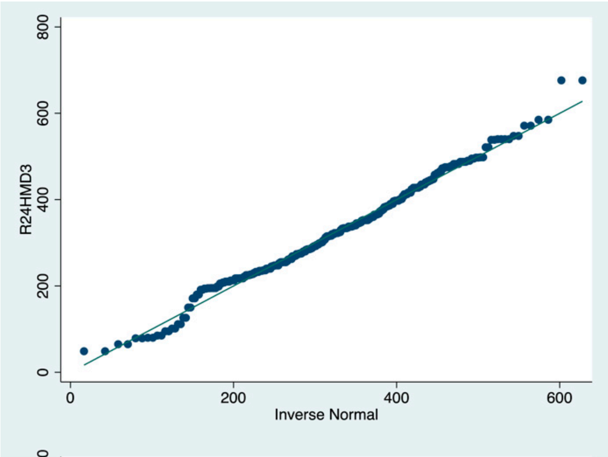
R24HMD3
0 200 400 600 800
Inverse Normal
0 200 400 600

i

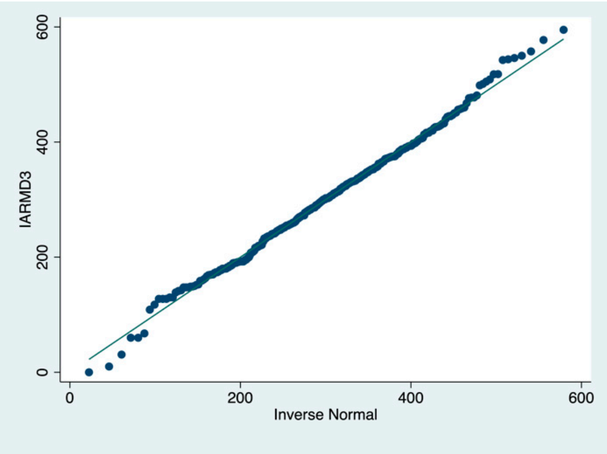
IARMD3
0 200 400 600
Inverse Normal
0 200 400 600

j

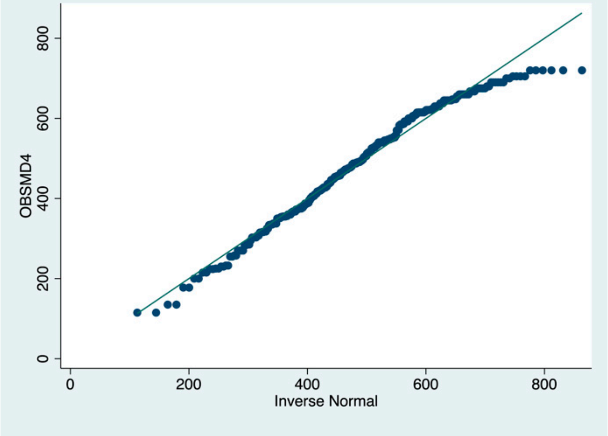
OBSMD4
0 200 400 600 800
Inverse Normal
0 200 400 600 800

k

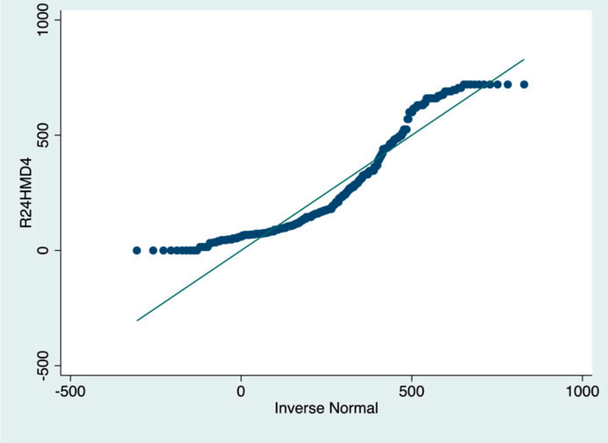
R24HMD4
-500 0 500 1000
Inverse Normal
-500 0 500 1000

l

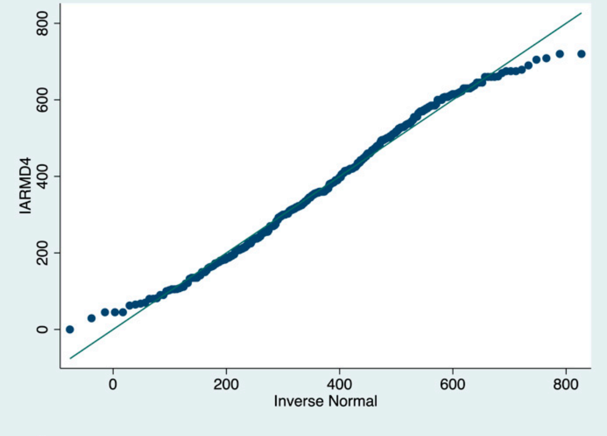
IARMD4
0 200 400 600 800
Inverse Normal
0 200 400 600 800

m

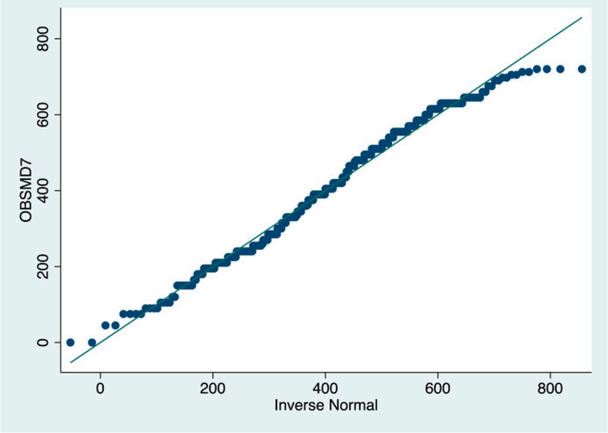
OBSMD7
0 200 400 600 800
Inverse Normal
0 200 400 600 800

n

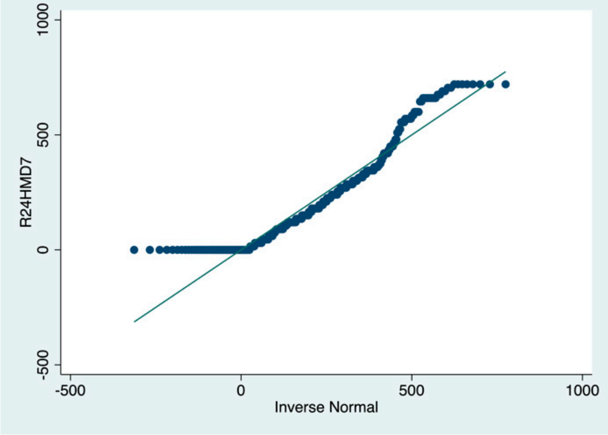
R24HMD7
-500 0 500 1000
Inverse Normal
-500 0 500 1000

o

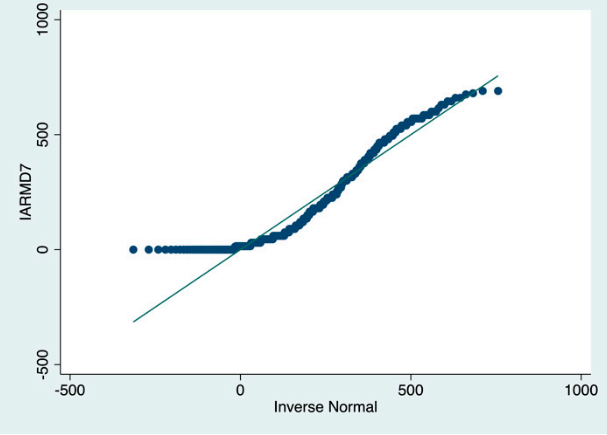
IARMD7
-500 0 500 1000
Inverse Normal
-500 0 500 1000

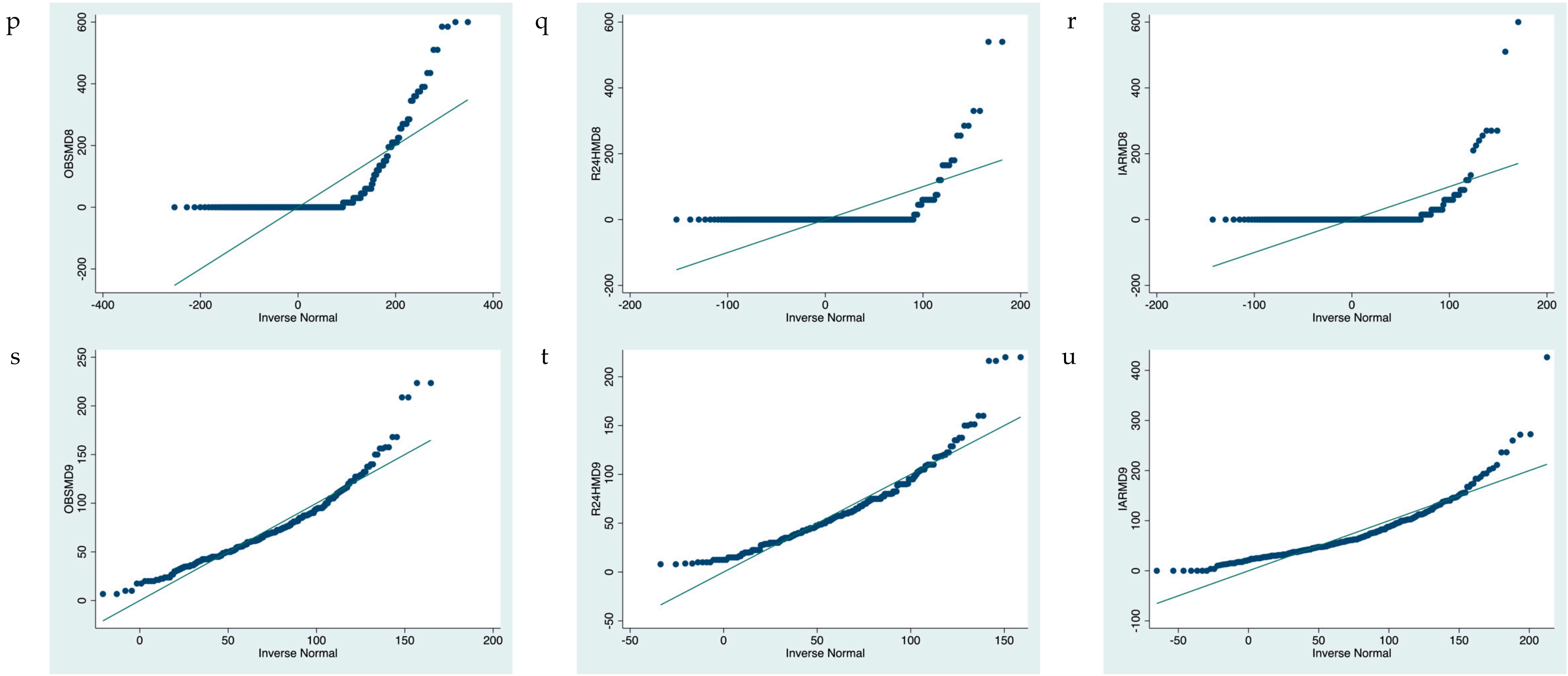

Figure S1. Plots of the quantiles of activities against the quantiles of the normal distribution (Q-Q plot) for employment and related activities [MD1] (a) OBS, (b) 24HR, and (c) IAR; production of goods for own final use [MD2]] (c) OBS, (e) 24HR, and (f) IAR; unpaid domestic services for household and family members [MD3]] (g) OBS, (h) 24HR, and (i) IAR; unpaid caregiving services for household and family members [MD4]] (j) OBS, (k) 24HR, and (l) IAR; socializing and communication, community participation and religious practice [MD7]] (m) OBS, (n) 24HR, and (o) IAR; culture, leisure, mass media and sports practice [MD8]] (p) OBS, (q) 24HR, and (r) IAR; and self-care and maintenance [MD9]] (s) OBS, (t) 24HR, and (u) IAR

SUPPLEMENTARY TABLES

Table S1. Household data collection patterns.

Pattern	DAY 1	DAY 2	DAY 3	DAY 4	DAY 5
**1**	ICF<br>MQ<br>Anthro	OBS<br>AWC	24HR<br>IAR	AWC	IAR<br>TQ
**2**	ICF<br>MQ<br>Anthro	AWC	IAR	OBS<br>AWC	24HR<br>IAR<br>TQ

ICF, informed consent form; MQ, mothers' questionnaire; Anthro, anthropometry; OBS, observation; AWC, automated wearable camera; 24HR, 24-hour recall; IAR, image-assisted recall; TQ, technology questionnaire.

Table S2. Time use activities and ICATUS major divisions.

ICATUS-2016 Major Divisions*		1<br>Employment and related activities	2<br>Production of goods for own final use	3<br>Unpaid domestic services for household and family	4<br>Unpaid caregiving services for household and family	5<br>Unpaid volunteer, trainee and other unpaid work †	6<br>Learning	7<br>Socializing and communication, community participation	8<br>Culture, leisure, mass media and sports practices	9<br>Self-care and maintenance
1	Cash crop farming	X								
2	Cooking food - business	X								
3	Working – employed	X								
4	Working - own business	X								
5	Supervising employees	X								
6	Food crop farming		X							
7	Fish farming		X							
8	Livestock rearing		X							
9	Fish-livestock product processing		X							
10	Shelling maize – hand		X							
11	Shelling maize –machine		X							
12	Wild food gathering & fishing		X							
13	Fetching fuel (incl. travel)		X							
14	Fetching water (incl. travel)		X							
15	Chopping / splitting firewood			X						
16	Other domestic work			X						
17	Other shopping			X						
18	Washing clothes			X						
19	Cooking food - family / friends			X						
20	Food collection – garden / farm			X						
21	Food collection – shop / vendor			X						
22	Food preparation			X						
23	Other post-harvest processing			X						
24	Travel to / from farm - food			X						
25	Travel to / from market - food			X						
26	Other travel			X						
27	Care of index‡				X					
28	Care of other children or adults‡				X					
29	Accessing services				X					
30	Breast feeding index				X					
31	Feeding index food or drinks				X					
32	Feeding other children or adults				X					
33	Playing w index				X					
34	Studying						X			
35	Chatting w friends / relatives‡							X		
36	Religious activities							X		
37	Social activities & hobbies							X		
38	TV/radio/reading‡								X	
39	Personal care									X
40	Eating or drinking									X
41	Resting§									
42	Sleeping§									
43	Answering the phone for the study§									
44	Other interactions for the study§									

* United Nations Statistics Division. (2019). International Classification of Activities for Time-Use Statistics 2016 (ICATUS 2016). New York: United Nations. † There were no activities that mapped to ICATUS MD5, Unpaid volunteer, trainee and other unpaid work. ‡ Simultaneous activity. § The following activities were not included in the analysis: resting or sleeping (due to known inconsistencies with recording) or answering the phone for the study or other interactions for the study.

Table S3. Inter-method comparison of the median time allocated in minutes to activity groups, including enumerator image interpretation (EII). (Median value, and 25th and 75th percentiles)

ICATUS Activity Group	OBS (N=175)		24HR (N=175)				EII (N=175)				IAR (N=175)			
	Median (minutes)	25th, 75th	Median (minutes)	25th, 75th	MD	P	Median (minutes)	25th, 75th	MD	P	Median (minutes)	25th, 75th	MD	P
Employment and related activities [MD1]	0	0, 5	0	0, 0	0	0.7987	0	0, 0	0	0.6050	0	0, 35	0	0.1123
Production of goods for own final use [MD2]	45	10, 79	49	15, 90	-3	0.0043	35	0, 75	5	0.0230	43	18, 81	0	0.8619
Unpaid domestic services for household and family members [MD3]	318	263, 370	320	245, 396	6	0.7903	295	218, 355	25	<0.0001	311	251, 374	8	0.1836
Unpaid caregiving services for household and family member [MD4] *	491	388, 608	180	96, 390	264	<0.0001	315	208, 445	153	<0.0001	418	324, 541	56	<0.0001
Socializing and communication, community participation and religious practice [MD7] *	405	270, 525	195	75, 330	180	<0.0001	90	15, 225	225	<0.0001	285	105, 465	105	<0.0001
Culture, leisure, mass media and sports practices [MD8] *	0	0, 30	0	0, 0	0	<0.0001	0	0, 0	0	<0.0001	0	0, 0	0	<0.0001
Self-care and maintenance [MD9]	68	50, 88	58	39, 80	11	0.0010	49	31, 78	14	<0.0001	79	53, 111	-9	0.0002

OBS, observation; 24HR, 24-hour recall; EII, enumerator image interpretation; IAR, image-assisted recall; MD, median of the time allocation differences (versus OBS); P, p-value of Wilcoxon signed rank sum test of time allocated compared to OBS.

* Activity group contains one or more simultaneous activities.

Table S4. Inter-method comparison of the median time allocated in minutes to activity groups *for participating women only*. (Median value, and 25th and 75th percentiles)

**ICATUS Activity Group**	**N**	**OBS**		**24HR**		**IAR**	
		Median (minutes)	25th, 75th	Median (minutes)	25th, 75th	Median (minutes)	25th, 75th
Employment and related activities [MD1]	77	16	0, 64	10	0, 60	53	4, 135
Production of goods for own final use [MD2]	159	50	15, 83	60	26, 94	45	24, 85
Culture, leisure, mass media and sports practices [MD8] *	73	45	15, 165	0	0, 0	0	0, 60

OBS, observation; 24HR, 24-hour recall; IAR, image-assisted recall.

* Activity group contains one or more simultaneous activities.

NB: There were no activities that mapped to ICATUS MD5, Unpaid volunteer, training, or other unpaid work.

NB: This table does not include resting or sleeping (due to known inconsistencies with recording) or answering the phone for the study or other interactions for the study.

Table S5. Inter-method comparison of the median number of concurrent activities. (Median value, and 25th and 75th percentiles)

	N	OBS		24HR		IAR	
		Median	25th, 75th	Median	25th, 75th	Median	25th, 75th
Median concurrent activities	175	3.0	2.5, 4.0	2.0†	1.0, 2.0	3.0	2.0, 4.0

OBS, observation; 24HR, 24-hour recall; IAR, image-assisted recall.

† P-value of Wilcoxon signed rank sum test of median time allocated compared to OBS <0.05.

NB: This data reflects the medians across the 40 discrete activities, not (the 9) activity groups.

NB: This table does NOT include resting or sleeping (due to known inconsistencies with recording) or answering the phone for the study or other interactions for the study.

Table S6. Inter-method comparison of the median number and proportion of timeslots containing concurrent activities. (Median value and percent)

Timeslots	OBS	24HR	IAR
Median number (out of 48)	42	26	40
Median proportion	88%	54%	83%

OBS, observation; 24HR, 24-hour recall; IAR, image-assisted recall.

Table S7. Inter-method comparison of the median time allocated in minutes to activity groups, observation day verses non-observation day. (Median value, and 25th and 75th percentiles)

**ICATUS Activity Group**	**NON OBS EII (N=174)**		**OBS EII (N=175)**		**P**	**NON OBS IAR (N=174)**		**OBS IAR (N=175)**		**P**
	Median	25th, 75th	Median	25th, 75th		Median	25th, 75th	Median	25th, 75th	
Employment and related activities [MD1]	0	0, 8	0	0, 0	0.0691	0	0, 26	0	0, 35	0.6984
Production of goods for own final use [MD2]	53	8, 104	35	0, 75	0.0005	60	16, 109	43	18, 81	0.0021
Unpaid domestic services for household and family members [MD3]	267	188, 345	295	218, 355	0.0525	296	224, 376	311	251, 374	0.0678
Unpaid caregiving services for household and family member [MD4] *	235	140, 398	315	208, 445	0.0014	339	206, 480	418	324, 541	<0.0001
Socializing and communication, community participation & religious practice [MD7] *	150	45, 285	90	15, 225	0.0141	278	150, 450	285	105, 465	0.3588
Culture, leisure, mass media and sports practices [MD8] *	0	0, 0	0	0, 0	0.6070	0	0, 0	0	0, 0	0.4107
Self-care and maintenance [MD9]	49	28, 79	49	31, 76	0.4350	74	44, 110	79	53, 111	0.2469

NON-OBS EII, non-observation day enumerator image interpretation; OBS EII, observation day enumerator image interpretation; P, p-value of Wilcoxon signed rank sum test of time allocated, non-observation day compared to observation day; NON-OBS IAR, non-observation day image-assisted recall; OBS IAR, observation day image-assisted recall.
* Activity group contains one or more simultaneous activities.

Table S8. Inter-method comparison of the median time allocated in minutes to activity groups, observation day verses non-observation day for households having IAR administered before OBS only. (Median value, and 25th and 75th percentiles)

**ICATUS Activity Group**	**NON OBS EII (N=88)**		**OBS EII (N=88)**		**P**	**NON OBS IAR (N=88)**		**OBS IAR (N=88)**		**P**
	Median	25th, 75th	Median	25th, 75th		Median	25th, 75th	Median	25th, 75th	
Employment and related activities [MD1]	0	0, 0	0	0, 0	0.5538	0	0, 20	0	0, 24	0.9906
Production of goods for own final use [MD2]	50	15, 111	36	2, 74	0.0020	59	23, 108	42	19, 79	0.0127
Unpaid domestic services for household and family members [MD3]	273	208, 335	299	224, 353	0.2973	314	233, 390	307	244, 370	0.7487
Unpaid caregiving services for household and family member [MD4] *	225	144, 390	345	228, 441	0.0020	327	213, 466	427	337, 558	<0.0001
Socializing and communication, community participation & religious practice [MD7] *	128	45, 263	60	15, 219	0.1031	270	143, 473	248	90, 420	0.2164
Culture, leisure, mass media and sports practices [MD8] *	0	0, 0	0	0, 0	0.2837	0	0, 0	0	0, 0	0.3081
Self-care and maintenance [MD9]	49	25, 75	53	34, 78	0.2243	66	47, 102	78	58, 114	0.0609

NON-OBS EII, non-observation day enumerator image interpretation; OBS EII, observation day enumerator image interpretation; P, p-value of Wilcoxon signed rank sum test of time allocated, non-observation day compared to observation day; NON-OBS IAR, non-observation day image-assisted recall; OBS IAR, observation day image-assisted recall.
* Activity group contains one or more simultaneous activities.

Table S9. Inter-method comparison of the median time allocated in minutes to activity groups, observation day verses non-observation day for households having IAR administered after OBS only. (Median value, and 25th and 75th percentiles)

ICATUS Activity Group	NON OBS EII (N=86)		OBS EII (N=87)		P	NON OBS IAR (N=86)		OBS IAR (N=87)		P
	Median	25th, 75th	Median	25th, 75th		Median	25th, 75th	Median	25th, 75th	
Employment and related activities [MD1]	0	0, 23	0	0, 8	0.0521	0	0, 31	0	0, 62	0.5889
Production of goods for own final use [MD2]	58	5, 95	35	0, 83	0.0735	62	5, 110	44	18, 84	0.0613
Unpaid domestic services for household and family members [MD3]	257	173, 364	285	218, 365	0.0827	288	203, 342	314	255, 375	0.0050
Unpaid caregiving services for household and family member [MD4] *	264	128, 405	270	188, 445	0.1591	340	198, 503	414	316, 529	0.0044
Socializing and communication, community participation & religious practice [MD7] *	150	30, 345	105	30, 225	0.0770	300	165, 435	300	180, 480	0.9639
Culture, leisure, mass media and sports practices [MD8] *	0	0, 0	0	0, 0	0.7003	0	0, 0	0	0, 0	0.8926
Self-care and maintenance [MD9]	49	28, 80	48	30, 68	0.8413	77	37, 125	79	50, 102	0.8955

NON-OBS EII, non-observation day enumerator image interpretation; OBS EII, observation day enumerator image interpretation; P, p-value of Wilcoxon signed rank sum test of time allocated, non-observation day compared to observation day; NON-OBS IAR, non-observation day image-assisted recall; OBS IAR, observation day image-assisted recall.

* Activity group contains one or more simultaneous activities.

Table S10. Frequency of inter-method time allocation differences.

**ICATUS Activity Group**	Method	Frequency of no differences N (%)	Frequency of differences <15 min N (%)	Frequency of differences <30 min N (%)	Frequency of differences >1 hr N (%)	Frequency of differences >2 hr N (%)
Employment and related activities [MD1]	24HR	114 (65)	126 (72)	138 (79)	26 (15)	11 (6)
	IAR	105 (60)	118 (67)	132 (75)	23 (13)	8 (5)
Production of goods for own final use [MD2]	24HR	26 (15)	74 (42)	111 (63)	27 (15)	6 (3)
	IAR	29 (17)	84 (48)	121 (69)	17 (10)	5 (3)
Unpaid domestic services for household and family members [MD3]	24HR	1 (1)	20 (11)	43 (25)	98 (56)	46 (26)
	IAR	1 (1)	29 (17)	54 (31)	73 (42)	19 (11)
Unpaid caregiving services for household and family member [MD4] *	24HR	0 (0)	7 (4)	9 (5)	159 (91)	139 (79)
	IAR	1 (1)	10 (6)	25 (14)	125 (71)	84 (48)
Socializing and communication, community participation and religious practice [MD7] *	24HR	5 (3)	5 (3)	10 (6)	151 (86)	134 (77)
	IAR	4 (2)	4 (2)	12 (7)	144 (82)	112 (64)
Culture, leisure, mass media and sports practices [MD8] *	24HR	109 (62)	109 (62)	123 (70)	37 (21)	25 (14)
	IAR	110 (63)	110 (63)	125 (71)	36 (21)	22 (13)
Self-care and maintenance [MD9]	24HR	1 (1)	52 (30)	97 (55)	19 (11)	4 (2)
	IAR	2 (1)	46 (26)	96 (55)	30 (17)	11 (6)

* Activity group contains one or more simultaneous activities.

Table S11. Frequency of inter-method median concurrent activities differences.

	Method	Frequency of no differences N (%)	Frequency of differences <1 CA N (%)	Frequency of differences <2 CA N (%)	Frequency of differences >4 CA N (%)
Median concurrent activities	24HR	30 (17)	43 (25)	92 (53)	1 (1)
	IAR	37 (21)	55 (31)	129 (74)	8 (5)

CA, concurrent activities; 24HR, 24-hour recall; IAR, image-assisted recall.

Table S12. Inter-method comparison of the median concurrent activities bias and limits of agreement (LOA).

	Bias* (activities)	LOA†	
24HR	1.3	-1.1	3.7
IAR	0.0	-3.2	3.2

LOA, limits of agreement; 24HR, 24-hour recall; IAR, image-assisted recall.
* Mean difference.
† +/- 2 SD from the mean difference.
NB: A negative indicates that 24R / IAR overestimated OBS.

Table S13. Inter-method comparison of reliability for median concurrent activities.

	**24HR**					**IAR**				
	% Agree.	Cohen's kappa†	P	95% CI		% Agree.	Cohen's kappa†	P	95% CI	
Median concurrent activities	0.7091	0.0281	0.198	-0.0149	0.0710	0.8521	0.0308	0.487	-0.0565	0.1181

24HR, 24-hour recall; IAR, image-assisted recall; CI, confidence interval.
† Using weighted Cohen's (reliability) kappa. Landis and Koch (1977) suggest the following benchmark scale for interpreting the kappa statistic: <0.00 Poor; 0.00-0.20 Slight; 0.21-0.40 Fair; 0.41-0.60 Moderate; 0.61-0.80 Substantial; 0.81-1.00 Almost Perfect.

Table S14. Inter-method comparison of the median time allocated in minutes to discrete activities. (Median value, and 25th and 75th percentiles)

		OBS		24HR				EII				IAR			
	N	*Median*	*25th, 75th*	*Median*	*25th, 75th*	*MD*	*P*	*Median*	*25th, 75th*	*MD*	*P*	*Median*	*25th, 75th*	*MD*	*P*
**ICATUS MD1 - Employment and related activities**															
Cash crop farming	175	0	0, 0	0	0, 0	0	0.7550	0	0, 0	0	0.0334	0	0, 0	0	0.2040
Cooking food - business	175	0	0, 0	0	0, 0	0	0.2328	0	0, 0	0	0.0009	0	0, 0	0	0.0067
Working – employed	175	0	0, 0	0	0, 0	0	0.9968	0	0, 0	0	0.1573	0	0, 0	0	0.1573
Working - own business	175	0	0, 0	0	0, 0	0	0.8234	0	0, 0	0	0.0616	0	0, 35	0	0.0003
Supervising employees	175	0	0, 0	0	0, 0	0	0.0833	0	0, 0	0	0.0833	0	0, 0	0	0.0833
**ICATUS MD2 - Production of goods for own final use**															
Food crop farming	175	0	0, 0	0	0, 0	0	0.1136	0	0, 15	0	0.0740	0	0, 0	0	0.5500
Fish farming	175	0	0, 0	0	0, 0	0	0.3173	0	0, 0	0	.	0	0, 0	0	.
Livestock rearing	175	0	0, 5	0	0, 0	0	0.4705	0	0, 0	0	<0.0001	0	0, 8	0	0.9048
Fish-livestock product processing	175	0	0, 0	0	0, 0	0	0.6491	0	0, 0	0	0.1573	0	0, 0	0	0.9954
Shelling maize – hand	175	0	0, 0	0	0, 0	0	0.9968	0	0, 0	0	0.1573	0	0, 0	0	0.1573
Shelling maize –machine	175	0	0, 0	0	0, 0	0	0.1831	0	0, 0	0	0.0455	0	0, 0	0	0.1764
Wild food gathering & fishing	175	0	0, 0	0	0, 0	0	0.3173	0	0, 0	0	0.3173	0	0, 0	0	0.3173
Fetching fuel (incl. travel)	175	0	0, 8	0	0, 0	0	0.4273	0	0, 0	0	0.0003	0	0, 10	0	0.8883
Fetching water (incl. travel)	175	20	0, 45	28	0, 60	0	0.0069	13	0, 43	0	0.2054	20	0, 45	0	0.8087
**ICATUS MD3 - Unpaid domestic services for household and family members**															
Chopping / splitting firewood	175	0	0, 8	0	0, 0	0	<0.0001	0	0, 0	0	<0.0001	0	0, 0	0	0.0014
Other domestic work	175	83	50, 114	53	23, 90	27	<0.0001	58	25, 83	23	<0.0001	68	34, 98	19	<0.0001
Other shopping	175	0	0, 0	0	0, 0	0	0.1508	0	0, 0	0	0.0013	0	0, 0	0	0.0137
Washing clothes	175	0	0, 20	0	0, 15	0	0.9219	0	0, 23	0	0.0165	0	0, 26	0	0.6586
Cooking food - family / friends	175	85	56, 111	100	60, 158	-15	0.0001	64	38, 96	20	0.0001	70	48, 100	13	0.0023
Food collection – garden / farm	175	0	0, 15	0	0, 15	0	0.2274	0	0, 0	0	0.0002	0	0, 15	0	0.9843
Food collection – shop / vendor	175	0	0, 0	0	0, 0	0	0.6527	0	0, 0	0	0.0013	0	0, 5	0	0.0755
Food preparation	175	51	30, 74	45	15, 73	4	0.1814	48	23, 78	0	0.9477	49	26, 79	1	0.9976
Other post-harvest processing	175	0	0, 13	0	0, 0	0	<0.0001	0	0, 24	0	0.0023	0	0, 28	0	<0.0001
Travel to / from farm - food	175	0	0, 20	0	0, 15	0	0.6650	0	0, 0	0	0.0056	0	0, 13	0	0.0352
Travel to / from market - food	175	0	0, 0	0	0, 0	0	0.7101	0	0, 0	0	0.5086	0	0, 11	0	0.0003
Other travel	175	11	0, 33	0	0, 23	0	0.0259	15	0, 53	0	0.0017	15	0, 53	0	0.0043
**ICATUS MD4 - Unpaid caregiving services for household and family members**															
Care of index *	175	405	285, 510	105	30, 300	225	<0.0001	240	135, 360	120	<0.0001	315	210, 465	60	<0.0001
Care of other children or adults *	175	255	105, 390	30	0, 150	150	<0.0001	120	45, 255	90	<0.0001	165	45, 270	45	<0.0001
Accessing services	175	0	0, 0	0	0, 0	0	0.6585	0	0, 0	0	0.0254	0	0, 0	0	0.9943
Breast feeding index	175	44	0, 78	0	0, 23	15	<0.0001	0	0, 0	26	<0.0001	0	0, 45	0	<0.0001
Feeding index food or drinks	175	35	21, 57	20	10, 33	14	<0.0001	31	15, 50	4	0.1069	46	28, 70	-9	0.0001
Feeding other children or adults	175	21	10, 33	15	5, 26	6	0.0001	19	8, 33	0	0.6409	25	11, 38	-2	0.1400

		OBS		24HR				EII				IAR			
	N	*Median*	*25th, 75th*	*Median*	*25th, 75th*	*MD*	*P*	*Median*	*25th, 75th*	*MD*	*P*	*Median*	*25th, 75th*	*MD*	*P*
Playing w index	175	15	0, 35	0	0, 0	13	<0.0001	0	0, 0	13	<0.0001	0	0, 15	8	<0.0001
**ICATUS MD6 - Learning**															
Studying	175	0	0, 0	0	0, 0	0	.	0	0, 0	0	.	0	0, 0	0	.
**ICATUS MD7 - Socializing and communication, community participation and religious practice**															
Chatting w friends / relatives *	175	390	270, 525	180	75, 315	195	<0.0001	90	15, 225	225	<0.0001	270	105, 450	105	<0.0001
Religious activities	175	0	0, 0	0	0, 0	0	0.2514	0	0, 0	0	0.0254	0	0, 0	0	0.7202
Social activities & hobbies	175	0	0, 0	0	0, 0	0	0.2302	0	0, 0	0	0.0044	0	0, 0	0	0.5385
**ICATUS MD8 - Culture, leisure, mass media and sports practices**															
TV/radio/reading *	175	0	0, 30	0	0, 0	0	<0.0001	0	0, 0	0	<0.0001	0	0, 0	0	<0.0001
**ICATUS MD9 - Self-care and maintenance**															
Personal care	175	30	19, 45	26	8, 38	8	0.0024	0	0, 13	23	<0.0001	29	15, 49	1	0.4473
Eating or drinking	175	35	24, 48	30	19, 45	3	0.0819	38	24, 63	-6	0.0071	40	28, 63	-6	<0.0001

OBS, observation; 24HR, 24-hour recall; EII, enumerator image interpretation; IAR, image-assisted recall; MD, mean proportion difference (versus OBS); P, p-value of Wilcoxon signed rank sum compared to OBS.

* Simultaneous activity.

NB: There were no activities that mapped to ICATUS MD5, Unpaid volunteer, trainee and other unpaid work.

NB: This table does not include resting or sleeping (due to known inconsistencies with recording) or answering the phone for the study or other interactions for the study.

Table S15. Within-method comparison of time allocation - with and without simultaneous activities.

ICATUS Activity Group	Allowing for SA OBS		Traditional Approach OBS		% Change	Allowing for SA 24HR		Traditional Approach 24HR		% Change	Allowing for SA IAR		Traditional Approach IAR		% Change
	Median (minutes)	25th, 75th	Median (minutes)	25th, 75th		Median (minutes)	25th, 75th	Median (minutes)	25th, 75th		Median (minutes)	25th, 75th	Median (minutes)	25th, 75th	
Employment and related activities [MD1]	0	0, 5	0	0, 3	0%	0	0, 0	0	0, 0	0%	0	0, 35	0	0, 24	0%
Production of goods for own final use [MD2]	45	10, 79	26	6, 43	-42%	49	15, 90	38	8, 75	-22%	43	18, 81	29	11, 61	-33%
Unpaid domestic services for household and family members [MD3]	318	263, 370	185	143, 223	-42%	320	245, 396	250	180, 318	-23%	311	251, 374	208	168, 275	-33%
Unpaid caregiving services for household and family member [MD4]	491	388, 608	274	211, 341	-44%	180	96, 390	140	75, 223	-22%	418	324, 541	237	194, 299	43%
Socializing and communication, community participation and religious practice [MD7]	405	270, 525	137	89, 165	-66%	195	75, 330	108	38, 169	-47%	285	105, 465	97	39, 140	-66%
Culture, leisure, mass media and sports practices [MD8]	0	0, 30	0	0, 8	0%	0	0, 0	0	0, 0	0%	0	0, 0	0	0, 0	0%
Self-care and maintenance [MD9]	68	50, 88	41	31, 57	-40%	58	39, 80	48	30, 68	-17%	79	53, 111	57	40, 82	-28%

OBS, observation; 24HR, 24-hour recall; EII, enumerator image interpretation; IAR, image-assisted recall; MD, mean proportion difference (versus OBS); P, p-value of Wilcoxon signed rank sum compared to OBS.
